# E2SGAN: EEG-to-SEEG translation with generative adversarial networks

**DOI:** 10.3389/fnins.2022.971829

**Published:** 2022-09-01

**Authors:** Mengqi Hu, Jin Chen, Shize Jiang, Wendi Ji, Shuhao Mei, Liang Chen, Xiaoling Wang

**Affiliations:** ^1^School of Computer Science and Technology, East China Normal University, Shanghai, China; ^2^Institute for Biomedical Informatics, University of Kentucky Lexington, Lexington, KY, United States; ^3^Department of Neurosurgery, Huashan Hospital of Fudan University, Shanghai, China

**Keywords:** EEG-SEEG mapping, GANs, epilepsy, signal synthesis, stereoelectroencephalography, deep learning

## Abstract

High-quality brain signal data recorded by Stereoelectroencephalography (SEEG) electrodes provide clinicians with clear guidance for presurgical assessments for epilepsy surgeries. SEEG, however, is limited to selected patients with epilepsy due to its invasive procedure. In this work, a brain signal synthesis framework is presented to synthesize SEEG signals from non-invasive EEG signals. First, a strategy to determine the matching relation between EEG and SEEG channels is presented by considering both signal correlation and spatial distance. Second, the EEG-to-SEEG generative adversarial network (E2SGAN) is proposed to precisely synthesize SEEG data from the simultaneous EEG data. Although the widely adopted magnitude spectra has proved to be informative in EEG tasks, it leaves much to be desired in the setting of signal synthesis. To this end, instantaneous frequency spectra is introduced to further represent the alignment of the signal. Correlative spectral attention (CSA) is proposed to enhance the discriminator of E2SGAN by capturing the correlation between each pair of EEG and SEEG frequencies. The weighted patch prediction (WPP) technique is devised to ensure robust temporal results. Comparison experiments on real-patient data demonstrate that E2SGAN outperforms baseline methods in both temporal and frequency domains. The perturbation experiment reveals that the synthesized results have the potential to capture abnormal discharges in epileptic patients before seizures.

## 1. Introduction

Stereoelectroencephalography (SEEG) is an intracranial recording that can pinpoint the areas of the brain where seizures occur (Chabardes et al., 2018). SEEG signals are acquired by depth electrodes implanted into the brain (Li et al., 2018). Its high spatial and temporal resolution enable the recording of high-amplitude and high-frequency intracranial discharges that are sometimes difficult to observe on scalp electroencephalogram (EEG) (Ramantani et al., 2016). Despite its enormous benefits, SEEG is yet to be a panacea. To implant the electrodes, an invasive surgical procedure is required to make 10–20 small incisions in the scalp and skull. SEEG is only reserved for selected epilepsy patients due to the potential risk of infection (Cossu et al., 2005).

EEG, on the other hand, is an electrophysiological recording of electrical activity on the scalp by placing electrodes in fixed positions (Henry, 2006). EEG is non-invasive, relatively safe, inexpensive, functionally fast, and has been widely used to observe the spontaneous electrical activity of the brain. The electromagnetic fields recorded by EEG represent the linear summation of collective source activity (Plummer et al., 2008; He et al., 2019). Nevertheless, its relatively low signal-to-noise ratio, due to the attenuation by the layers lying around the brain, hinders the use of EEG for accurate epilepsy diagnoses.

**Figure 1:**
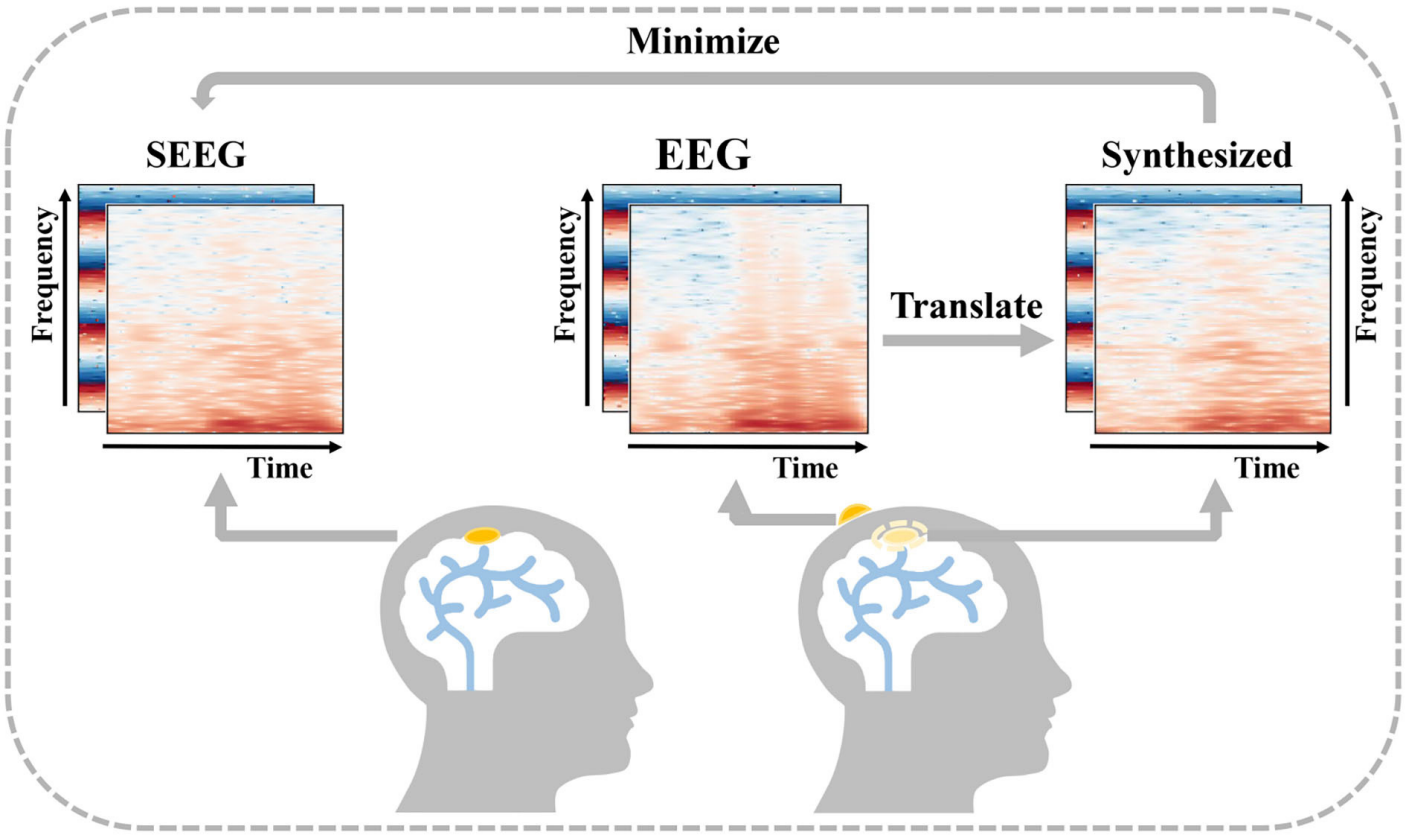
Description of EEG-to-Stereoelectroencephalography (SEEG) translation. EEG and SEEG are represented with a concatenated matrix of frequency and IF spectra. The difference between the real invasive SEEG and that synthesized from simultaneous non-invasive EEG is minimized.

In order to obtain intracranial signal recordings at low risk, a feasible solution is to recover an intracranial signal from a low-cost non-invasive signal. Cao et al. (2022) introduce the concept of virtual intracranial EEG (ViEEG) to reconstruct electrocorticography (ECoG) from magnetoencephalographic imaging (MEG). Dynamical network models are then applied to ViEEG to probe the underlying mechanisms of complex neural dynamics. Compared with ECoG, SEEG acts as an intracranial signal in the same way with richer spatial resolution, and the reconstruction is more challenging as well. As a collection of intracranial signals in the scalp, EEG is thought to be closely related to SEEG (Ramantani et al., 2016). Inspired by previous work and supported by the existing medical background, we propose a solution to synthesize intracranial SEEG from non-invasive EEG to face the above challenge and define this challenge as EEG-to-SEEG translation which is shown in Figure 1. The synthesized SEEG should retain the key features of real SEEG. In particular, the key features should carry clinical implications that can be regarded as plausible explanations of specific intracranial electrophysiological activity such as abnormal epileptic discharges. By indicating under what conditions key features are captured, clinicians can use the synthesized results in a targeted manner when assessing the need for SEEG implantation and then pinpointing the location for electrode implantation.

In recent years, the thriving of deep learning drives the development of various fields including EEG analysis, providing us with a new and feasible way of thinking. Antoniades et al. (2018) made an attempt to devise an asymmetric auto-encoder stacked with multi-layer neurons to map the temporal sequence of EEG to SEEG and outperform the previous linear methods such as least-squares regression (Kaur et al., 2014) and coupled dictionary learning (Spyrou and Sanei, 2016). The stacked architecture enhances the model's capacity but the simple auto-encoder architecture is still not powerful enough to achieve the desired result. Their attempt demonstrated the possibility of synthesizing SEEG from an input EEG and helped us recognize the necessity of a more powerful feature extractor and a sophisticated generation architecture. More recently, generative adversarial networks (GANs) (Goodfellow et al., 2014) have become a milestone in data generation and attracted our interest. GANs are basically composed of a generator network and a discriminator network. The process of adversarial training forces the generator to synthesize results with more details. As a result, the discriminator can hardly distinguish the real and generated ones. The ability of GANs to fit input and output distributions makes it outstanding in heterogeneous data synthesis (Jiao et al., 2019; Selim et al., 2020). The excellence of GANs soon inspired researchers to leverage this architecture as a new fashion to generate EEG signals (Hartmann et al., 2018; Luo T-j. et al., 2020; Yao et al., 2020). Furthermore, an improvement in GAN by imposing a condition on the input has achieved great success in image-to-image translation (Isola et al., 2017), which makes it possible to transfer the style or texture of the input to the output image. They utilized PatchGAN as a new paradigm of discriminator in order to restrict GANs to only model high-frequency structures. Their work enlightened us to leverage conditional GANs (cGANs) (Mirza and Osindero, 2014) together with PatchGAN-based paradigm to transfer an EEG segment to the SEEG segment.

Although the above methods are mature and proved to be effective, most of them only consider temporal representation, neglecting the informative features hidden behind. Numerous studies have demonstrated that time-frequency representations obtained from Short-Time Fourier Transform (STFT) (Li et al., 2021) or Morlet wavelet convolutions provide richer information and help give better predictions (Yao et al., 2018; Wang et al., 2020). Clinically, different EEG bands have discriminative implications (Tatum, 2014) and serve as beneficial features in many tasks (Yao et al., 2018; Wang et al., 2020). Therefore, in the context of EEG-to-SEEG translation, it is necessary to explore and exploit the correlation between two signals from a frequency domain perspective. Another discovery that has intrigued us is that the partial derivative of the unwrapped phase with respect to time, commonly referred to as the instantaneous frequency (IF), has great potential in the synthesis of phase spectra (Engel et al., 2019; Marafioti et al., 2019). Better phase spectrum synthesis ensures more coherent temporal results, which is one of the aims of this work.

Furthermore, SEEG electrodes outnumber EEG electrodes in most cases, and the placement of SEEG electrodes varies from patient to patient. Therefore, for the set-wise translation, it is inevitable to determine the matching relationship between the EEG and the SEEG set. This requires us to develop a strategy to select reasonable pairs from a large number of candidate fragments. The selected pairs are expected to contain clinically meaningful features. Subject to the complexity of EEG data, we were unable to accurately capture key signals such as potential pre-seizure micro-abnormal discharges. However, based on the good synchronization property of EEG and SEEG, it can be assumed that this key signal must be hidden in pairs with a strong correlation. In general, this correlation is affected by physical distance, and the strength can be measured by calculations based on power spectral density.

In this work, by leveraging both the temporal and frequency characteristics of brain signals, an EEG-SEEG matching strategy is designed to construct an aligned dataset and an EEG-to-SEEG generative adversarial network called E2SGAN is proposed.

First, the EEG-SEEG matching strategy explores the nonlinear correlation between EEG and SEEG by observing how signal similarity varies with spatial distance. An aligned dataset is constructed with the selected pairs in the form of the time-frequency representation obtained from the STFT transform.

Second, the E2SGAN trained on the aligned dataset is proposed to convert the input EEG to the corresponding SEEG. The E2SGAN architecture takes full advantage of the time-frequency features and learns how to synthesize the magnitude and IF spectra accurately. The generator is built with residual blocks connecting a CNN-based encoder-decoder structure, and the discriminator is designed according to the patch-based paradigm. Two auxiliary modules, called correlative spectral attention (CSA) and weighted patch prediction (WPP), are devised to enhance the discriminator's ability. CSA captures the correlation between different combinations of EEG and SEEG frequencies and prevents the discriminator from making judgments based solely on the geometry of the spectra. WPP is a technique that eliminates potential mode collapse that occurs with each frequency to ensure more robust temporal results.

Extensive comparison experiments have shown that the proposed framework is able to outperform the baseline methods. The perturbation experiment reveals that the synthesized results have the potential to capture abnormal discharges in epileptic patients before seizures.

The main contributions of this work are as follows:

- We propose E2SGAN, a practical deep-learning algorithm to address the EEG-to-SEEG translation. CSA and WPP are devised to capture the correlation between EEG and SEEG spectra and ensure robust temporal results.
- We pioneer the introduction of both magnitude and IF spectra as a time-frequency representation in a brain signal conversion setup.
- We develop an EEG-SEEG matching strategy to determine the matching relation between EEG and SEEG sets. The strategy explores the nonlinear correlation of signal similarity with respect to spatial distance.
- Evaluation results on extensive real-patient-based experiments demonstrate the excellent performance of the proposed framework in both temporal and frequency domains. A further perturbation experiment reveals the potential of the synthesized results to capture abnormal epileptic discharges.

## 2. Materials and methods

### 2.1. Framework overview for SEEG synthesis

**Figure 2:**
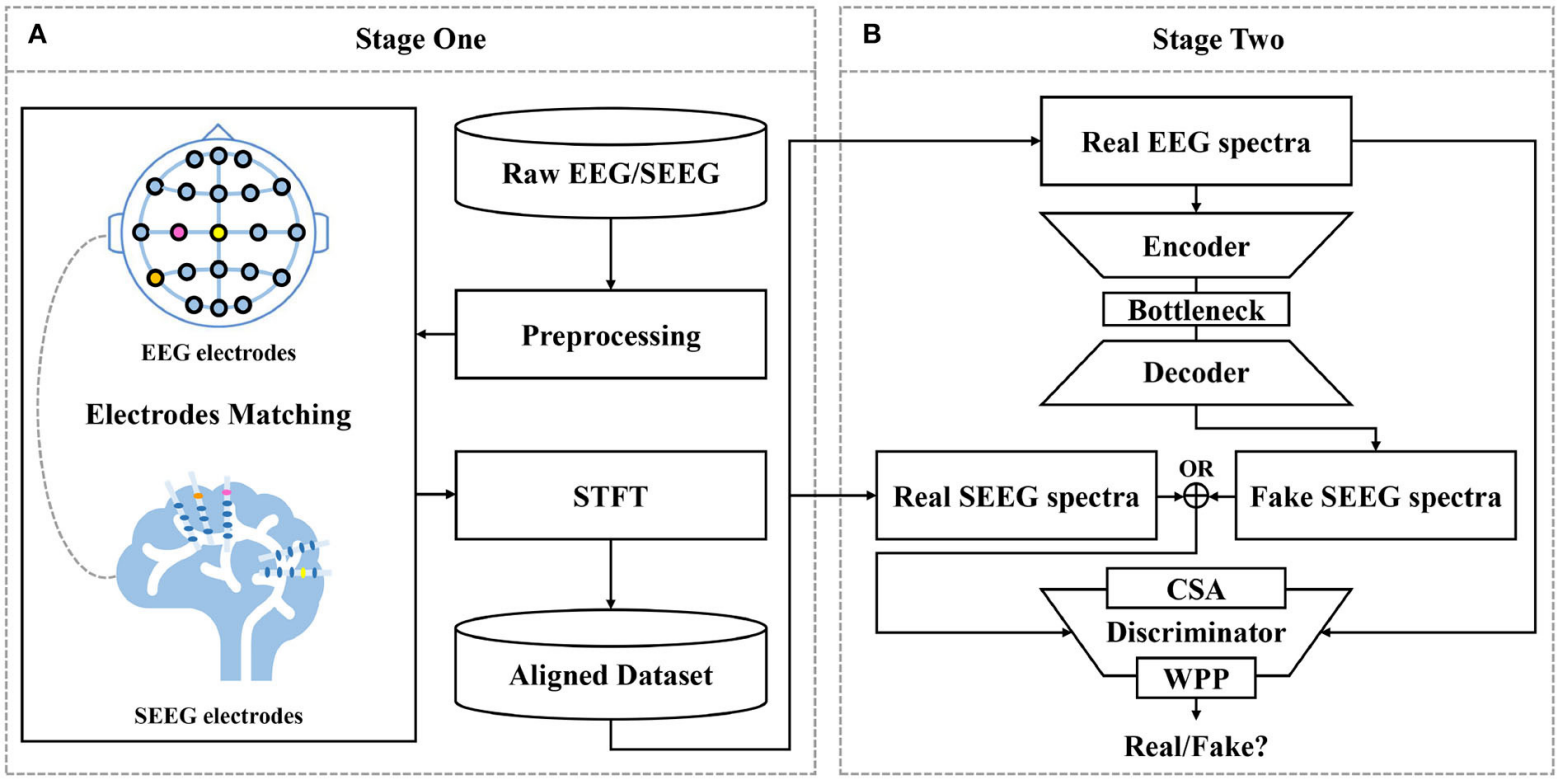
Proposed framework for EEG-to-SEEG translation. **(A)** Raw EEG and SEEG signals are filtered and segmented using a synchronized sliding window. Then, segments from two sources are matched into pairs with the proposed strategy. Short-Time Fourier Transform (STFT) is performed to obtain magnitude and IF spectra. The aligned dataset is constructed with the processed pairs. **(B)** EEG-to-SEEG generative adversarial network (E2SGAN) is trained on the aligned dataset to synthesize SEEG from simultaneous EEG. Correlative Spectral Attention (CSA) and Weighted Patch Prediction (WPP) are devised to give a further boost to the discriminator.

A two-fold pipeline is designed for SEEG synthesis as depicted in Figure 2, (1) to prepare aligned training data by matching EEG and SEEG segments (see Section 2.2) and (2) to translate EEG to SEEG (see Section 2.3 to 2.3.6).

The first stage does a preliminary job of organizing preprocessed EEG and SEEG signals in pairs to form a temporally aligned dataset. Raw EEG and SEEG signals are filtered and segmented using a synchronized sliding window. Then, segments from two sources are matched into pairs with the proposed matching strategy (see Section 2.2) based on the correlation between signal similarity and physical distance. The paired segments are processed with STFT to obtain magnitude spectra and IF spectra. The aligned dataset is constructed with the processed pairs.

At the second stage, E2SGAN is trained on the aligned dataset. The generator transforms the input EEG spectra to the target SEEG spectra while the discriminator makes an effort to distinguish real and fake targets conditioned by the input EEG. CSA (see Section 2.3.4) and WPP (see Section 2.3.5) are two auxiliary modules devised to give a further boost to the discriminator. Specifically, CSA is a mutual attention sub-network that captures the correlation between EEG and SEEG frequencies. The captured correlation can be considered as an extra supervision signal which ensures the correct frequency correlation of the synthesized target with respect to the input. WPP is a customized technique to disturb the monotonous distribution of patch prediction. The variation within patch-based prediction alleviates the mode collapse caused by low variance in IF spectra. The optimization of the whole network is described in Section 2.3.6. In the end, the synthesized SEEG spectra are transformed back to temporal representation *via* inverse STFT. The implementation of the proposed framework is available at https://anonymous.4open.science/r/E2SGAN-180B/.

### 2.2. EEG-SEEG matching strategy

As is discussed in Section 1, a matching strategy aims to address two challenges. First, it has to make a compromise on the difficulty of directly translating the whole set of EEG to SEEG. Second, it should single out the potential pairs carrying implicit clinical features, which can be measured by the strength of correlation within a pair. Therefore, we settle for the second best to focus on one-to-one mapping within pairwise EEG and SEEG channels. Based on this setting, the set-wise translation is decomposed to sub-tasks where the pairwise translation will be performed. Such a strategy has to guarantee the existence of a correlation between EEG and SEEG segments within a pair. Specifically, a qualified solution should obey the following procedure:

1. Map the set of EEG channels to the set of SEEG channels *via* any form of bipartite graph matching to obtain a sea of candidate EEG-SEEG pairs
2. Search for the optimal pairs while ensuring the correlation
3. Generalize the solution so as to be applied to upcoming subjects

**Figure 3:**
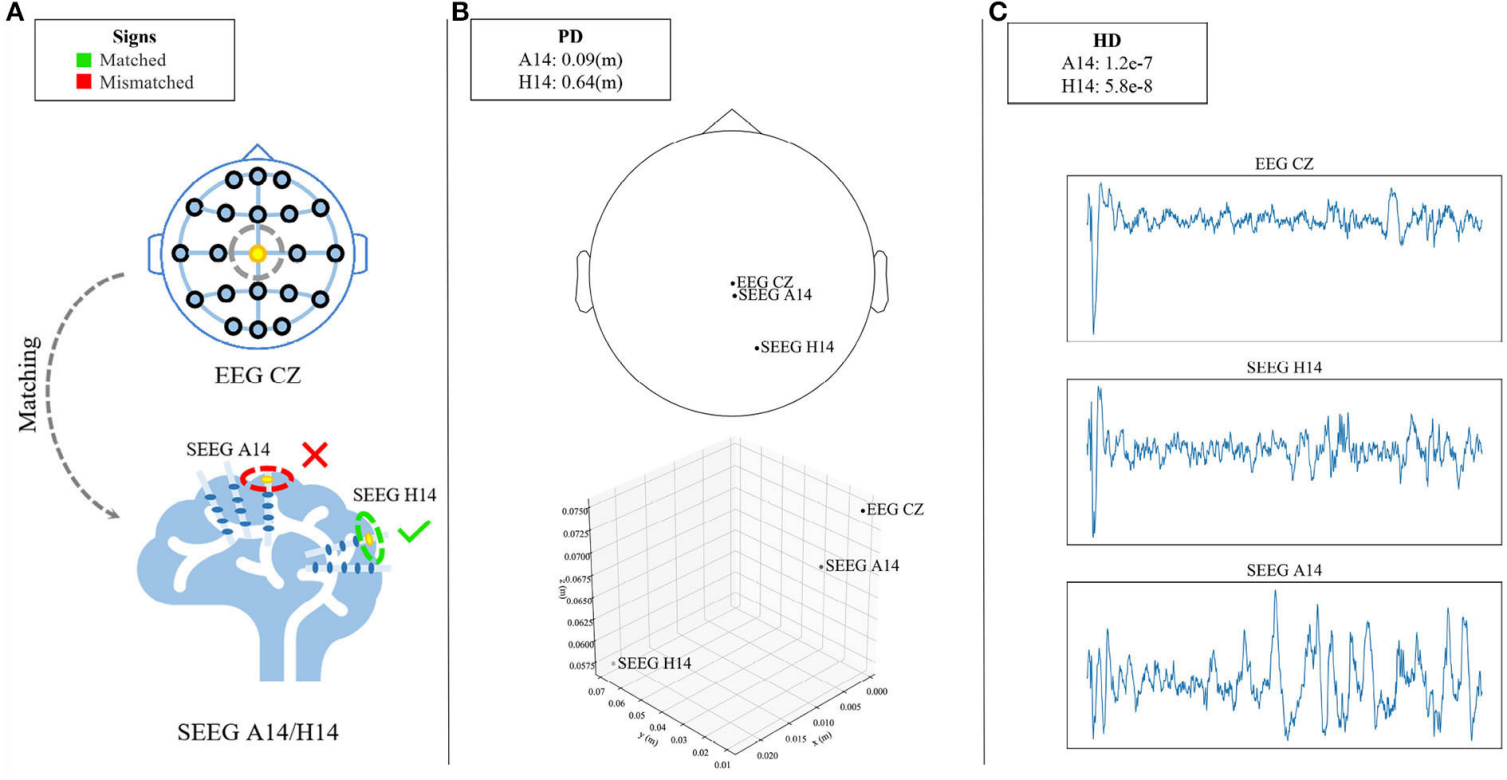
Demonstration of the counterintuitive relation between signal correlation and physical distance. **(A)** Illustrates that the matching strategy needs to match the EEG channel (*Cz*, yellow-doted at the top) to one of the candidate SEEG channels (*A14*/*H14*, red-/green-circled at the bottom). Despite the closer physical distance of SEEG *A14* to EEG *Cz* which is shown in the topological map (top of **B**) and the 3-D location (bottom of **B**), the farther HD is observed in *A14* rather than *H14* **(C)**. *H14* is considered to be an appropriate match to *Cz*.

For this strategy, such correlation is defined as signal similarity based on a given similarity metric. The correlation does not vary with distance in a linear trend as is habitually deemed, but rather a non-linear fashion. To demonstrate this counterintuitive relation, the Hellinger Distance (Chen et al., 2020) from two SEEG channels and the target EEG channel are compared as shown in Figure 3. The leftmost column gives an example of what a matching strategy is expected to solve. When matching the EEG *Cz* (yellow-dotted at the top of Figure 3A), the strategy is making a decision between the choice of SEEG *A14* and *H14* (red-/green-circled at the bottom of Figure 3A). Despite the closer physical distance of the SEEG channel *A14* to EEG *Cz* as is shown in the topological map (top of Figure 3B) and the 3-D location (bottom of Figure 3B), the farther HD is observed in *A14* rather than *H14* in Figure 3C. Hence, *H14* is considered to be an appropriate match to *Cz*. Potential reasons can be the influence of brain geometry or brain functional connectivity on signal propagation (Frauscher et al., 2018). To guarantee a meaningful pairwise translation, a matching strategy has to take into account the complex correlation within a pair.

Here, one of the implementations following the proposed procedure is presented in Algorithm 1 which deals with the complex correlation in a simple but efficient way. Regarding the EEG and SEEG channels as two non-overlapped sets, the problem is initiated as bipartite graph matching. To fulfill Procedure 1, all the SEEG channels are first matched to a given EEG channel to obtain candidate EEG-SEEG pairs. In Algorithm 1, Euclidean distance is adopted as *d*(·) to sort the pairs. Considering the non-linear trend, the set of *C* is divided into subsets, leaving each subset corresponding with a distance interval *itv*.

**Algorithm 1:**
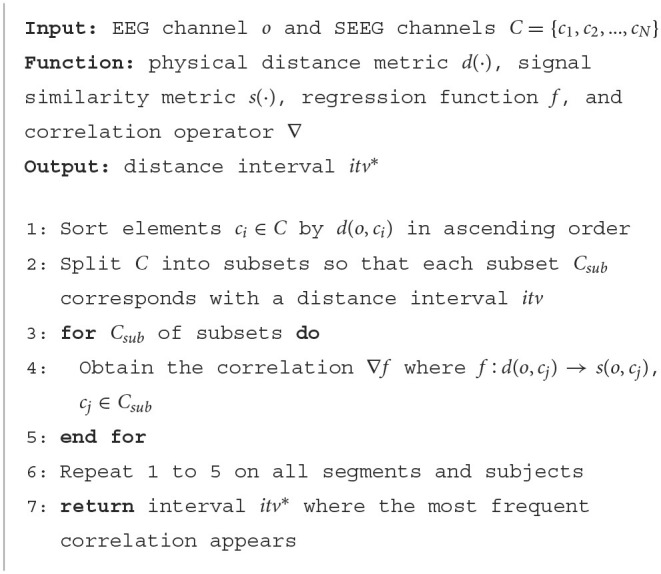
EEG-SEEG Matching Strategy

For each subset, linear regression is used as *f* to explore the correlation between physical distance and signal similarity. Here, Hellinger Distance is adopted as *s*(·) and the first-order derivative ∇*f* is calculated, of which the positive value indicates that the similarity between the EEG channel and SEEG channels decreases as the physical distance becomes farther. The proportion of positive ∇*f* is counted on each subset. The steps mentioned above are carried out on all segments and subjects. After that, the interval itv^*^ where the most positive derivatives appear, ranging from *a* to *b* mm, i.e., itv^*^ = [*a, b*], is chosen. SEEG channel approximately *a* mm away from the investigated EEG is considered a match.

Within itv^*^, the similarity between EEG and SEEG segments prominently declines as the physical distance increases. Latent correlations between SEEG and EEG signals have been shown within this interval, even not necessarily stronger than the others, thus satisfying Procedure 2. Other off-the-shelf methods for finding optimal EEG-SEEG similarity can be directly applied to the proposed strategy by replacing the metric *s*(·) in Algorithm 1.

In the following experiments, EEG *Cz* is chosen to obtain the output interval itv^*^, and the conclusion is extended to all other EEG channels as is required by Procedure 3.

### 2.3. E2SGAN method

In the second stage, we center on the EEG to SEEG translation. In this section, some rudiments of instantaneous frequency and conditional GAN are provided as preliminary. Then, the details about the generative model are provided, including two auxiliary modules and the objectives.

#### 2.3.1. Brain signal representation with instantaneous frequency

To better characterize brain signals, we employ STFT, which transforms the original signal into the frequency domain for the generation task. Different from the previous classification tasks, in addition to considering the energy distribution of the brain signal in the frequency domain, the phase distribution of the signal needs to be taken into consideration. Otherwise, the time domain representation of the signal cannot be restored. In practice, frame-based techniques used in signal process/generation such as deconvolutions will cause the initial phase of the segment intercepted by the frame to change over time when the stride of frames does not equal the signal's periodicity. This phenomenon renders the generation of phase spectra a tricky task as covering all the necessary frequencies and all possible phase alignments to preserve the phase coherence is impractical (Engel et al., 2019).

The partial derivative of the unwrapped phase with respect to time, commonly referred to as instantaneous frequency (IF), is a time varying measure of the true signal oscillation. For example, given a function of phase with respect to time


(1) $$\begin{array}{l} \varphi \left(t\right)=\omega t+\theta \end{array}$$

where ω is the frequency and θ is the initial phase or phase offset. The instantaneous frequency is defined as


(2) $$\begin{array}{l} \omega \left(t\right)=\frac{d\varphi \left(t\right)}{dt} \end{array}$$

where φ(*t*) has to be in unwrapped form (Sejdic et al., 2008). In this case, a time-independent constant θ is derived. It has been proved that the instantaneous frequency of phase is a more promising modeling target than the phase itself when generating signals or phase spectra (Marafioti et al., 2019). The assumption of instantaneous frequency alleviates this risk since it remains constant on each frequency and is feasible to be learned by neural networks.

#### 2.3.2. Conditional GANs for EEG-to-SEEG translation

The Generative Adversarial Network proposed by Goodfellow et al. (2014) has been proved to have strong data generation ability, which benefits from its unique network architecture: a pair of generator network and discriminator network competing with each other to generate indistinguishable data. The generator network is usually composed of an encoder-decoder structure, which takes noise sampled from a known prior distribution as input and aims to fit the target data distribution as precisely as possible. The discriminator network focuses on determining whether the input data comes from the target data distribution or is forged by the generator. Analogous to the game process, the two networks optimize their own parameters through the feedback given by each other and finally produce an output that is indistinguishable from real and fake.

To control the modes of the data being generated, Mirza and Osindero (2014) proposed the conditional version of GANs. Given a generator network *G* and a discriminator network *D*, the optimization objective of cGANs can be formulated as follows:


(3) $$\begin{array}{l} L_{cGAN}=E_{x\sim p_{data}\left(x\right)}\left[\log D\left(x|y\right)\right]+E_{z\sim p_{z}\left(z\right)}\left[\log\left(1-D\left(G\left(z|y\right)\right)\right)\right] \end{array}$$

where ***x*** is a sample from the target distribution, ***y*** is the condition, and ***z*** is sampled from a prior distribution. The core idea of cGANs is to concatenate an extra condition to the inputs of both *G* and *D*, imposing the networks to determine whether the generated data matches the given condition. If ***y*** also conforms to a known distribution, we are able to realize the conversion between the two different data distributions. To ensure a one-to-one mapping, the addition of noise needs to be removed.

The basic idea of cGANs is adopted to achieve the goal of translating EEG to SEEG. Overall, the generator *G* establishes a mapping from an EEG segment *e* to an SEEG segment *s* where *e*, *s* ∈ ℝ^2×*m*×*n*^. The first dimension represents the concatenated magnitude and IF spectra. *m* and *n* are the numbers of frequencies and time steps after the STFT operation. Subsequently, *D* takes the (*e, s*) pair as input where *s* is either a real SEEG segment or generated and outputs a scalar to indicate the difference between the distributions of real and fake pairs. PatchGAN (Isola et al., 2017) is adopted as *D*, which is a fully convolutional neural network that penalizes structure at the scale of patches based on the Markov chain assumption. Noise added to the input is omitted since the task is a determinate one-to-one mapping.

**Figure 4:**
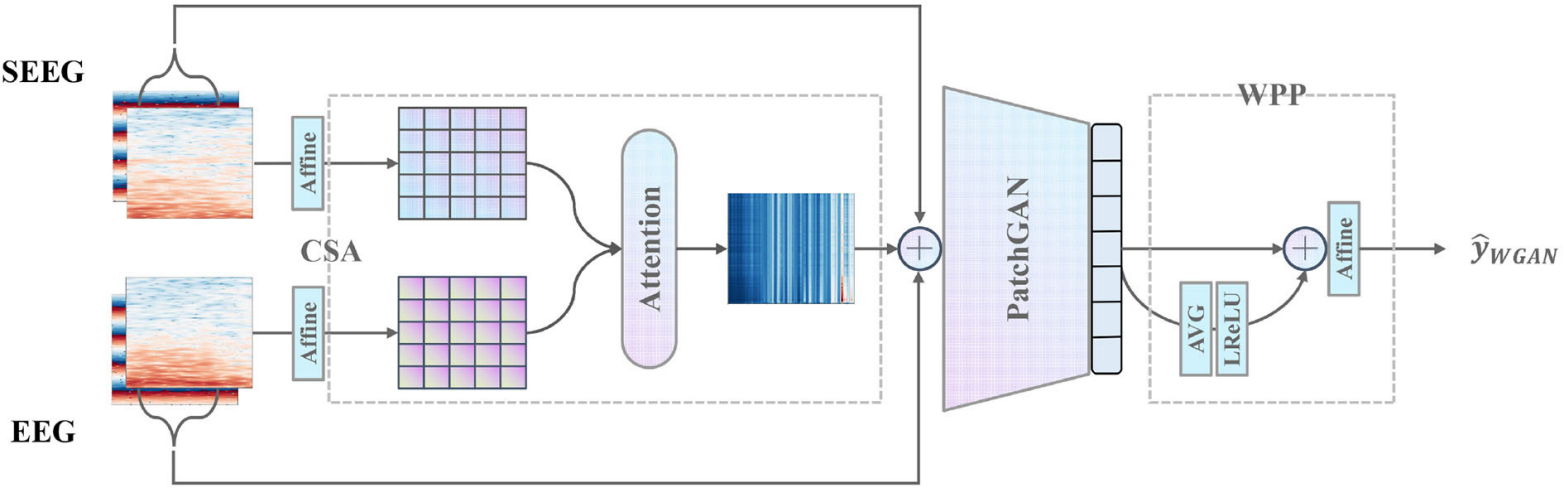
Proposed discriminator with the pre-positioned CSA module capturing the frequency correlation of EEG and SEEG, and the post-positioned WPP module ensuring robust temporal results.

Furthermore, CSA and WPP are devised to give a further boost to the discriminator as is shown in Figure 4. CSA is a mutual attention sub-network that captures the correlation between each combination of EEG and SEEG frequencies. The captured correlation can be considered an extra supervision signal which ensures the correct frequency correlation of the synthesized target with respect to the input. WPP is a customized technique to disturb the monotonous distribution of patch predictions. The variation within patch-based prediction alleviates the mode collapse caused by low variance in IF spectra and ensures robust temporal results.

#### 2.3.3. Architecture of generator and discriminator networks

The architectures of the generator and discriminator networks are shown in Table 1. The generator is basically a three-layer CNN autoencoder with two residual blocks as the bottleneck. 2D convolution filters are used for feature extraction since the input is similar to an image. It is worth mentioning that we use upsampling function followed by the same convolution instead of a deconvolution filter to avoid checkerboard artifacts (Odena et al., 2016). The discriminator is a PatchGAN, namely a fully-convolutional structure. Equalized learning rate layer and pixel-wise norm layer are applied to improve the stability of training (Karras et al., 2018). Leaky ReLU (Maas et al., 2013) is applied as the activation function.

**Table 1:** Architectures of the proposed generator and discriminator.

**Layers**	**Output shape**	**Norm./Act**.
**Generator**		
Input	2 ×128 ×128	–
Padding	2 ×128 ×128	ELR/LReLU/PN
Conv2d	32 ×64 ×64	ELR/LReLU/PN
Conv2d	64 ×32 ×32	ELR/LReLU/PN
ResBlock*2	64 ×32 ×32	ELR/LReLU/PN
Upsample	64 ×64 ×64	–
Conv2d	32 ×64 ×64	ELR/LReLU/PN
Upsample	32 ×128 ×128	–
Conv2d	16 ×128 ×128	ELR/LReLU/PN
Padding	16 ×134 ×134	–
Conv2d	2 ×128 ×128	Tanh
**Discriminator**		
Input	4 ×128 ×128	–
Conv2d	16 ×64 ×64	ELR/LReLU
Conv2d	32 ×32 ×32	ELR/LReLU
Conv2d	64 ×16 ×16	ELR/LReLU
Conv2d	128 ×15 ×15	ELR/LReLU
Conv2d	1 ×14 ×14	–

#### 2.3.4. Correlative spectral attention

To capture the latent correlation between the input EEG segment and the target SEEG segment, the CSA is proposed. It adopts a mutual attention module to model the correlation from a perspective of magnitude spectra. The mutual attention module serves as an observer that learns to express to what extent the correlation between EEG and SEEG segments is preserved and then passes the message to the discriminator as a supply.

Specifically, given an input pair (*e, s*), only the magnitude *E*_*mag*_ and $S_{mag}\in {\mathbb{R}}^{m\times n}$ are extracted. The magnitude is defined as a sequence of frequency vectors (*f*_1_, *f*_2_, ..., *f*_*m*_) where each frequency vector $f_{i}\in {\mathbb{R}}^{n}$ is a time series (*t*_1_, *t*_2_, ..., *t*_*n*_). To map *E*_*mag*_ and *S*_*mag*_ to a proper space, learned affine transformations *A*_1_ and *A*_2_ are applied. *A*(·, *w, b*) is defined as


(4) $$\begin{array}{l} A\left(X,w,b\right)=X\cdot w+b \end{array}$$

where *X* is the input matrix, *w* ∈ ℝ^*n*×*n*^ and *b* ∈ ℝ^*n*^ are learnable affine parameters. To further obtain the correlative expressions, mutual attention is calculated between *E*_*mag*_ and *S*_*mag*_. The attention score α is defined as


(5) $$\begin{array}{l} \alpha =softmax\left(\frac{A_{1}\left(E_{mag},w_{1},b_{1}\right)\cdot A_{2}{\left(S_{mag},w_{2},b_{2}\right)}^{\text{T}}}{\sqrt{n}}\right) \end{array}$$

where each scalar in α ∈ ℝ^*m*^ can be considered as the level of correlations between different combinations of EEG and SEEG frequencies. *E*_*mag*_ is multiplied by α to obtain the output of CSA


(6) $$\begin{array}{l} CSA\left(e,s\right)=\alpha \cdot E_{mag} \end{array}$$

and concatenate it with the original pair (*e, s*) as the final input to *D*. Here, the output of CSA acts as an auxiliary supervision signal which suggests the principal components of input correlated to the target. CSA reinforces the temporal-frequency representation to be better utilized by the downstream PatchGAN.

#### 2.3.5. Weighted patch prediction

The patch-based technique described in Isola et al. (2017) is not powerful enough to discriminate the features of an image in low variance situations since a little change in features provides many similar sub-images of monotonous mode and therefore leads to mode collapse. In practice, the low variance in IF along each frequency is observed, which leads to mode collapse in both generated magnitude and IF spectra. The collapsed spectra have a salient characteristic of repetitive stripes along with each frequency. To eliminate the undesirable results, a patch-weighting technique is adopted by applying a learnable weight to the prediction of a patch. The weighted patch predictions are then conforming to a distorted distribution distinguishable from each other. In addition, the averaged representation of all patch predictions is weighted to provide a global view of the input to adjust the final prediction.

Specifically, the original PatchGAN *D* outputs a one-channel matrix *P* ∈ ℝ^1 × *k*×*k*^, of which the element represents the prediction of a patch. *k* is the number of rows in the output matrix. *P* is flattened to obtain vector *p* ∈ ℝ^*k*^^2^. The global representation *p*_*global*_ is calculated by averaging the predictions in *p* and applying a non-linear transformation:


(7) $$\begin{array}{l} p_{global}=LReLU\left(\frac{1}{k^{2}}\overset{k^{2}}{\underset{i=1}{\sum }}p_{i}\right) \end{array}$$

We choose leaky rectified activation (LReLU) (Maas et al., 2013) as the non-linear function because it has been proved to work well for training GAN models (Radford et al., 2015). LReLU helps to ensure the gradient can flow through the entire architecture. Then, *p*_*global*_ and *p* are concatenated in preparation for the weighted prediction $\hat{y}$, which is defined as


(8) $$\begin{array}{l} q=cat\left(p_{global},p\right),q\in {\mathbb{R}}^{k^{2}+1} \end{array}$$

(9) $$\begin{array}{l} \hat{y}=\overset{k^{2}+1}{\underset{i=1}{\sum }}\beta_{i}q_{i} \end{array}$$

In Equation (9), β_*i*_ denotes the learnable weight assigned to the global view and the patch predictions. The behavior of weighting disturbs the original monotonous distribution among patches. The global representation *p*_*global*_ plays a role in providing the complete insight into the input since it collects views from all patches.

#### 2.3.6. Objectives

WGAN-GP (Gulrajani et al., 2017) is adopted to stabilize the training of cGAN. The objective with the proposed auxiliary modules are formulated as follows:


(10) $$\begin{array}{l} \begin{array}{l} L_{GAN}=E_{e,s\sim p_{r}\left(e,s\right)}\left[D\left(e,s,CSA\left(e,s\right)\right)\right]-\\ \;E_{e\sim p_{r}\left(e\right)}\left[D\left(e,G\left(e\right),CSA\left(e,G\left(e\right)\right)\right)\right]+\\ \;\lambda_{gp}E_{ê,ŝ\sim p_{i}\left(ê,ŝ\right)}\left[{\left(∥\nabla_{\left(ê,ŝ\right)}D\left(ê,ŝ,CSA\left(ê,ŝ\right)\right){∥}_{2}-1\right)}^{2}\right] \end{array} \end{array}$$

where *p*_*r*_(*e, s*) is the joint distribution of real pairs and *p*_*r*_(*e*) is the distribution of EEG. *p*_*i*_(ê, ŝ) is the joint distribution defined on the interpolated space derived from real and synthesized pairs. WGAN-GP uses the interpolated space derived from the real and synthesized samples to represent the input space of *D* in order to compute gradients. λ_*gp*_ is used to scale the gradient penalty.

L1 loss is adopted in the generator for it contributes to capturing the low-frequency components of a image (Isola et al., 2017), which in this work corresponds to the slow-changing regions of spectra:


(11) $$\begin{array}{l} \begin{array}{c} L_{L1}=E_{e,s\sim p_{r}\left(e,s\right)}\left[∥s-G\left(e\right){∥}_{1}\right] \end{array} \end{array}$$

As a result, the final min-max optimization objective is


(12) $$\begin{array}{l} G_{E2SGAN}^{*}=\arg\underset{G}{\;\min}\underset{{\;}_{D}}{\;\max\;}{ℒ}_{GAN}+\lambda_{L1}{ℒ}_{L1} \end{array}$$

where λ_*L*1_ is a hyper-parameter adjusting the L1 loss.

## 3. Results

### 3.1. Subjects and data recording

Seven subjects participated in the study. Subjects were patients with intractable epilepsy who had implanted SEEG electrodes for pre-surgical assessment of their seizure focus. All implant parameters were determined solely by clinical needs rather than those of this research. SEEG signals were acquired using a clinical recording system (EEG-1200C, Nihon Kohden, Irvine, CA) and sampled at 2,000 Hz. We also recorded scalp EEG simultaneously. All subjects gave informed consent to this study, which was approved by the Ethics Committee of Huashan Hospital (Shanghai, China).

### 3.2. Preprocessing

Both EEG and SEEG signals are vulnerable to noise interference. Slow signal drifts mask genuine cortical activity in the low frequency range (de Cheveigné and Arzounian, 2018). Electrical line noise causes unwanted effects on a fixed frequency of 50 or 60 Hz and their harmonics. To remove these unwanted components from the signal, EEG signal at 1 Hz and SEEG at 0.5 Hz is high-pass filtered to remove slow signal drifts (Li et al., 2018), and the signal at 50 Hz and their harmonics are notch filtered to remove line noise. SEEG electrodes located in the cortex are filtered out for the reason that they contributed little to the scalp signal. For the convenience of computation, the filtered signal is down-sampled to 64 Hz where the majority of power is distributed. Segmentation was performed on the continuous brain signals. A sliding window with a size of 1,016 sampling points (15.875 s) and a stride of a quarter of the window size is performed on the filtered signal. STFT with the window size of 256 and the hop length of 8 was performed to transform each segment to magnitude and phase spectra matrix with the same shape of (128, 128). IF spectra matrix from phase spectra is then derived. The final representation of each segment was a (2, 128, and 128) tensor.

We used pre-ictal EEG and SEEG recorded simultaneously from seven epileptic rest-state patients. The placement of EEG electrodes conformed to the 10–20 system. After segmentation and pairing, each subject had 8,000 EEG-SEEG pairs as the training set, 200 as the validation set, and 200 as the test set.

### 3.3. Evaluation metrics

The quality of results is evaluated by calculating the distance between real and generated SEEG segments from both temporal and frequency perspectives. From a temporal perspective, dynamic time warping (DTW) is used which matches two-time series through a dynamic programming strategy. From a frequency perspective, the Hellinger Distance and root mean square errors (RMSE) of the power spectral density (PSD) are adopted. The Hellinger Distance reflects the similarity of the power distribution of different frequencies. MSE of PSD reflects to what degree the dominant components of power are recovered. Inception score (Salimans et al., 2016) and Frechet Inception Distance (Heusel et al., 2017) are no longer considered in this work which is a determined mapping instead of generation from noise.

For evaluation, the leave-one-patient-out cross-validation strategy is employed. The averaged results are computed only on the test sets. Evaluation of the input EEG was used as a baseline. The evaluations are post-processed according to Equation (13) by dividing them by the baseline evaluation. A binary logarithm was taken as the final result. For each experiment, the baseline evaluation is shown on the header of the tables. For all metrics, a smaller value indicates a better result.


(13) $$\begin{array}{l} \begin{array}{c} Eval_{log}\left(S_{fake},S_{real}\right)=\log_{2}\frac{Eval\left(S_{fake},S_{real}\right)}{Eval\left(E_{real},S_{real}\right)} \end{array} \end{array}$$

### 3.4. Baselines

The proposed architecture is compared with different approaches using mainstream generative models. ASAE and AE adopt an encoder-decoder architecture while EEGGAN, GANSynth, and Pix2pix are based on GANs.

#### 3.4.1. ASAE

Asymmetric-Symmetric Autoencoder uses an asymmetric autoencoder to map scalp signal to intracranial signal, followed by a symmetric autoencoder to enhance the generated results.

#### 3.4.2. AE

This baseline has the same architecture as the generator in our method (see Table 1).

#### 3.4.3. EEGGAN

EEGGAN uses an improved WGAN-GP by gradually relaxing the gradient constraint to map a noise distribution to the EEG distribution. In the experiments, the input noise is replaced with EEG segments to generate SEEG.

#### 3.4.4. uTSGAN

uTSGAN uses two WGAN-GP sub-models to interactively learn the time- and frequency-domain generation of time series from a noise distribution. The input noise is replaced with reshaped EEG segments. It has to be mentioned that since the authors of uTSGAN did not open-source their code, we only reproduce their work in a simple way as efficiently as possible. We use 2D Conv for the spectral WGAN and 1D Conv for the temporal WGAN. The loss function and optimizers follow as described in the original work (Smith and Smith, 2020, 2021).

#### 3.4.5. Pix2pix

Pix2pix is a generic image-to-image translation approach based on cGANs. In the experiments, we implement the generator with the same architecture as the AE method and adopt PatchGAN which was originally used in the work (Isola et al., 2017).

#### 3.4.6. GANSynth

GANSynth is a GAN-based method used in audio synthesis which takes the magnitude and IF spectra as input to generate the audio segments in a progressive way. The input noise is also replaced with EEG segments.

For the baselines that are originally applied to signal generation such as ASAE, EEGGAN, and GANSynth, we follow the setting specified in the paper. In other words, STFT will not be performed to preserve the temporal representation for ASAE and EEGAN while magnitude and IF spectra are used for GANSynth. For the rest, magnitude and phase (instead of IF) spectra are used as a two-channel image to be compatible with the CNN-based architectures. For uTSGAN, we use both the temporal representation and the magnitude matrix.

### 3.5. Experiment settings

λ_*gp*_ and λ_*L*1_ are set to 10. The learning rate of *G* and *D* is set to 1e-5 and 2e-5, respectively. In the training phase, the model is first trained by 80 epochs without the CSA module and then jointly trained by another 40 epochs. We adopted Adam optimizer to perform gradient descent optimization (Kingma and Ba, 2014) and implemented our framework with Pytorch. Preprocessing is done with the help of the MNE toolkit (Gramfort et al., 2013).

### 3.6. Performance of different methods

#### 3.6.1. Comparison with baseline methods

The results of the proposed method trained on the aligned dataset guided by the proposed matching strategy introduced in Section 2.2 are denoted as E2SGAN-S. The comparison results are shown in Table 2. The full visual results are provided in Supplementary Figures 1, 2. ASAE performs poorly in DTW and HD because its encoder-decoder architecture simply based on stacks of fully-connected layers is not powerful enough to learn the complicated mapping between EEG and SEEG. Although it seems to perform well on PSD, the fact is that the model only has learned low-frequency pulses that dominate the magnitude of the power spectrum. AE is able to capture the majority of low-frequency features but underfits the high frequency. This reflects on the temporal result as slow fluctuations with simple ripples. Both the autoencoder-based methods overfit the low frequencies because of no extra regularization on high-frequency features. EEGGAN learns the mapping directly from the temporal domain and captures the general distribution of the target power spectrum, but it fails to learn the exact features of the waveform, which leads to a competitive performance only on HD. uTSGAN is inferior to the proposed E2SGAN and other baselines. Pix2pix has a slightly better performance compared to AE on PSD and HD. The patch-based discriminator enables it to learn more complicated patterns but also makes it hard to generate them accurately, resulting in poor performance on DTW. GANSynth achieves the most competitive performance among baselines because it utilizes IF spectra which contributes to the accuracy in phase recovery. The progressive approach enables it to capture the general power distribution and thus perform well on HD metrics. The proposed method achieves the best results on all metrics. The DTW result far superior to the others demonstrates the effectiveness of adopting IF spectra and the WPP technique. In addition, the lowest MSE of PSD and HD results suggest that the CSA module successfully captures not only the general power distribution of the target but also the exact magnitude of different frequencies by imposing regularization on the correlations between input and target frequencies.

**Table 2:** Performance comparison with baselines.

**Method**	**DTW/1.636e-3**	**PSD/1.128e-9**	**HD/2.886e-6**
ASAE (Antoniades et al., 2018)	2.867 ± 0.126	−1.384 ± 1.794	0.490 ± 0.800
AE^(*)^	−0.059 ± 0.815	−1.357 ± 1.659	0.337 ± 0.756
EEGGAN (Hartmann et al., 2018)	−0.019 ± 0.624	−0.821 ± 1.457	0.068 ± 0.734
uTSGAN (Smith and Smith, 2021)	3.867 ± 0.012	3.424 ± 0.134	3.127 ± 0.050
Pix2pix (Isola et al., 2017)	0.133 ± 0.829	−1.396 ± 1.754	0.316 ± 0.834
GANSynth (Engel et al., 2019)	−0.107 ± 0.740	−0.929 ± 1.453	0.191 ± 0.763
E2SGAN-N	**−0.424** **±** **0.400**	0.185 ± 0.298	0.572 ± 0.565
E2SGAN-S	−0.414 ± 0.764	**−1.480** **±** **1.609**	**−0.221** **±** **0.843**

AE^(*)^ is implemented with the same architecture as the generator of the proposed method. The bold values indicate the baseline values of evaluation.

#### 3.6.2. Influence of matching strategies

Furthermore, the performance of the proposed model on datasets constructed under the guidance of different matching strategies is investigated. A variant E2SGAN-N is proposed to be trained on the dataset guided by the nearest neighbor matching strategy. We adopt a most intuitive and practical strategy, that is to match the EEG and SEEG channels with the closest physical distance, and ensure the selection is not repeated. The results are shown at the bottom of Table 2. E2SGAN-N and E2SGAN-S have similar performance in the time domain, but E2SGAN-N is inferior to most baselines in the frequency domain. The main reason is that our proposed matching strategy explicitly guarantees the correlation between EEG-SEEG. This finding confirms the point mentioned in the previous Section 2.2, that the signals recorded by channels with closer distance do not necessarily have obvious correlations in the frequency domain. This can be mainly attributed to the irregular topology of gyri and sulci in the brain so that the propagation direction of intracranial signals does not necessarily follow the direction closest to the scalp. Assuming that the proposed model is sensitive to EEG-SEEG correlation, a paired dataset with a stronger correlation should achieve better performance. This experiment demonstrates the ability of E2SGAN to implicitly capture the correlation between EEG and SEEG.

### 3.7. Ablation study

In order to verify how CSA and WPP are beneficial to the whole model, three variants are compared to E2SGAN. For the “w/o WPP” variant, the WPP module is removed and the original PatchGAN prediction is used as output. For “w/o CSA” variant, we do not concatenate the CSA output to the input of PatchGAN. For “w/o CSA & WPP” variant, both of the aforementioned changes are adopted. As shown in Table 3, the removal of “WPP” degrades the performance in the temporal domain (DTW) and the removal of “CSA” degrades the performance in the frequency domain (PSD & HD). Although the two variants without “WPP” seem to perform better in the frequency domain, they actually suffer from mode collapse, leading to poor temporal robustness.

**Table 3:** Ablation study.

**Method**	**DTW/1.636e-3**	**PSD/1.128e-9**	**HD/2.886e-6**
w/o CSA & WPP	−0.318 ± 0.723	−1.633 ± 1.732	−0.393 ± 0.948
w/o WPP	−0.325 ± 0.729	**−1.681** **±** **1.734**	**−0.425** **±** **0.953**
w/o CSA	−0.346 ± 0.770	−1.385 ± 1.615	−0.112 ± 0.851
E2SGAN	**−0.414** **±** **0.764**	−1.480 ± 1.609	−0.221 ± 0.843

The bold values indicate the baseline values of evaluation.

#### 3.7.1. Temporal robustness study

**Figure 5:**
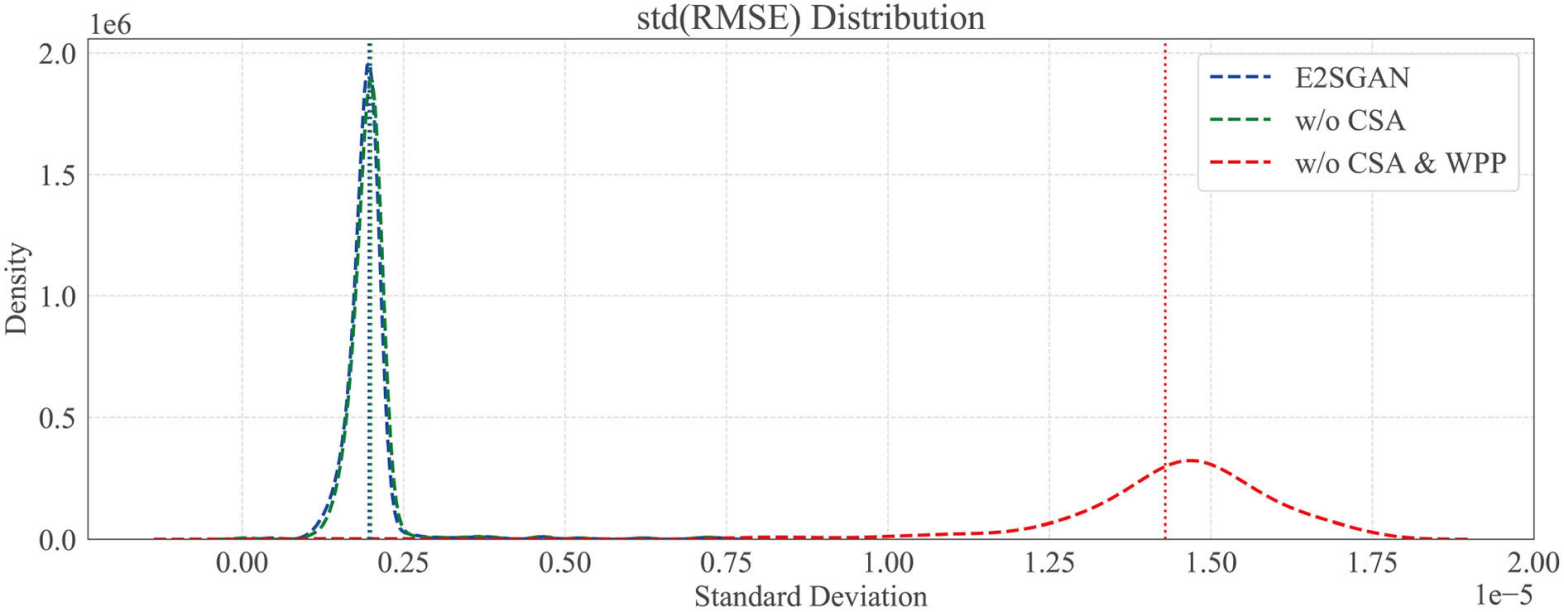
Temporal robustness study by comparing the standard deviation (SD) distribution of RMSE. Dotted vertical lines are the means of SD.

We further compare the proposed method to w/o CSA and w/o CSA & WPP in the temporal domain to verify the temporal robustness by calculating the standard deviation (STD) distribution of the RMSE between the real and synthesized SEEG. Specifically, each SEEG segment is equally divided into sub-segments, the width of which is traversed from 2 to half of the whole length. RMSE is calculated gradually on sub-segments, and the STD distribution of it on different scales of width is plotted in Figure 5. The horizontal axis is STD and the vertical axis is density. It can be clearly observed that the majority of STD of both methods with WPP are centrally distributed in low-value regions. The high STD in w/o CSA and WPP method implies some kind of spur occurring in the generated segments, which is actually observed in visual results.

### 3.8. Input-signal perturbation

To determine what key features in the input signal play a significant role in relating EEG to the simultaneous SEEG, input-signal perturbation is performed as is described in Gemein et al. (2020) and Schirrmeister et al. (2017). Specifically, random noise *z* ~ *N*(0, σ^2^) drawn from Gaussian distribution (with mean 0 and standard deviation σ identical with that of the original input) was added to the magnitude spectra of input EEG *e* while the phase was kept unperturbed. RMSE of PSD is computed with the perturbed EEG ê as input. Changes compared to the unperturbed result were computed to indicate the effect Δ_*effect*_ of perturbation.


(14) $$\begin{array}{l} \begin{array}{ll} \Delta_{effect}= & \sqrt{\frac{1}{m}\overset{m}{\underset{i=1}{\sum }}{\left(PSD{\left(G\left(ê\right)\right)}^{\left(i\right)}-PSD{\left(s\right)}^{\left(i\right)}\right)}^{2}}-\\ \; & \sqrt{\frac{1}{m}\overset{m}{\underset{j=1}{\sum }}{\left(PSD{\left(G\left(e\right)\right)}^{\left(j\right)}-PSD{\left(s\right)}^{\left(j\right)}\right)}^{2}} \end{array} \end{array}$$

Δ_*mag*_ denoting the changes in the magnitude of the input EEG was obtained by averaging the added noise *z*. Such perturbation was done on every EEG-SEEG pair and repeated 50 times on each investigated feature, including four frequency bands: δ (0–4 Hz), θ (4–7 Hz), α (8–15 Hz), β (16–31 Hz). For each feature, Δ_*effect*_ is correlated with Δ_*mag*_ by computing the correlation coefficient. It could be determined whether the increase or decrease in the magnitude of the investigated features contributed to a better or worse SEEG synthesis.

**Figure 6:**
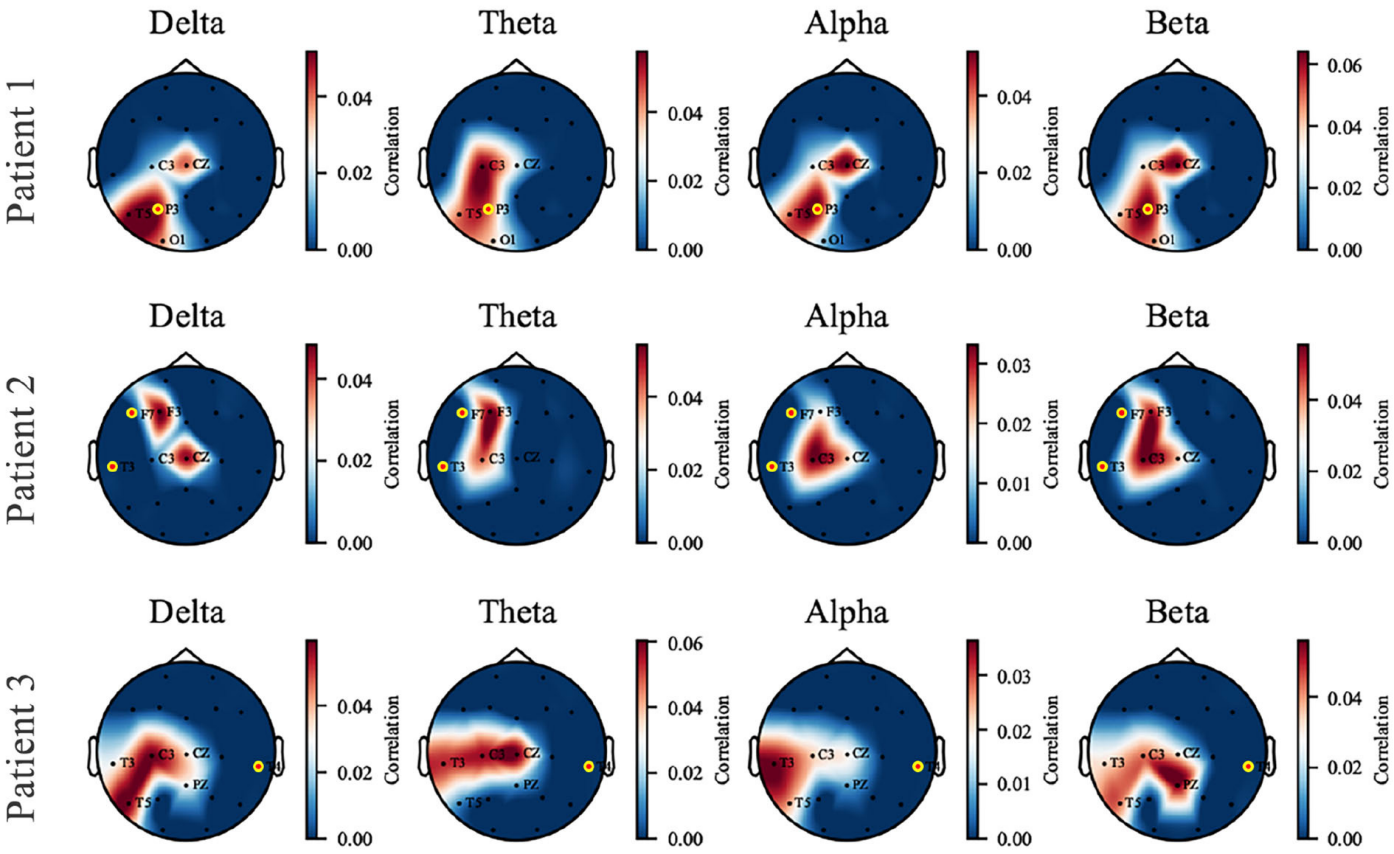
The topological map of correlation coefficient from input-signal perturbation where red indicates a high correlation and blue indicates a low correlation. Four bands (δ, θ, α, and β) are investigated on each patient, and three of them are displayed as representatives. The names of available EEG channels for each patient are listed, and the channels nearby a seizure focus are marked with yellow-edged dots. Patients 1 and 2 are good examples that the sensitive zone (left parietal and temporal zone for Patient 1, left frontal and temporal for Patient 2) coincides with the seizure location, while Patient 3 is an exceptional case due to the missing of *T4* channel in the dataset.

The perturbation operation is carried out individually on each patient. We selected three patients as representatives, who showed three kinds of responses to the proposed model to perturbation: (1) the captured perturbation-sensitive area is consistent with the epileptic focus; (2) the captured sensitive area is close to the focus; (3) being unable to capture sensitive areas near the focus due to data missing. The three cases are displayed in Figure 6 by visualizing the topological map of the correlation coefficients. EEG electrodes are partly missing due to the restriction in the collection and the proposed pairing strategy (which causes the unmatched channels to be discarded). We pay attention to the sensitivity of signals at different electrode locations to perturbation in different frequency bands. In patient 1, a dramatic effect is found in the parietal and temporal zones (*P3* and *T5*) of all bands and is most significant in the δ band. In patient 2, the most affected areas are the left frontal and central zones (*F3, C3*). In patient 3, the left temporal zone (*T3, T5*) is more sensitive. The added perturbation can be seen as a simulation of the underlying micro-abnormal discharges that occur before seizures. To verify that the sensitive channels we find are clinically significant, the seizure location provided by the clinician is marked with yellow-edged dots. Patient 1 shows that the seizure location (*P3*) coincides with the sensitive zone. For patient 2, the seizure location (*T3, F7*) is close to the sensitive zone we have found despite the missing of channels. An exceptional case is patient 3, where the real epileptic focus (*T4*) is in the right temporal zone, far away from the available channels in the training dataset. The cases of patients 1 and 2 demonstrate that the proposed model is able to capture the abnormal signal before seizures and is preserved in the synthesized SEEG. If the seizure location is too far away from the electrodes available in the dataset, our model will perform poorly. We provide the rest of the patients with Supplementary materials.

It has been verified that the δ power is associated with sleep and shows abnormal changes prior to seizures (Minecan et al., 2002; Long et al., 2021). The perturbation results in the δ band confirm the expected conclusion.

### 3.9. Case study of visual results

**Figure 7:**
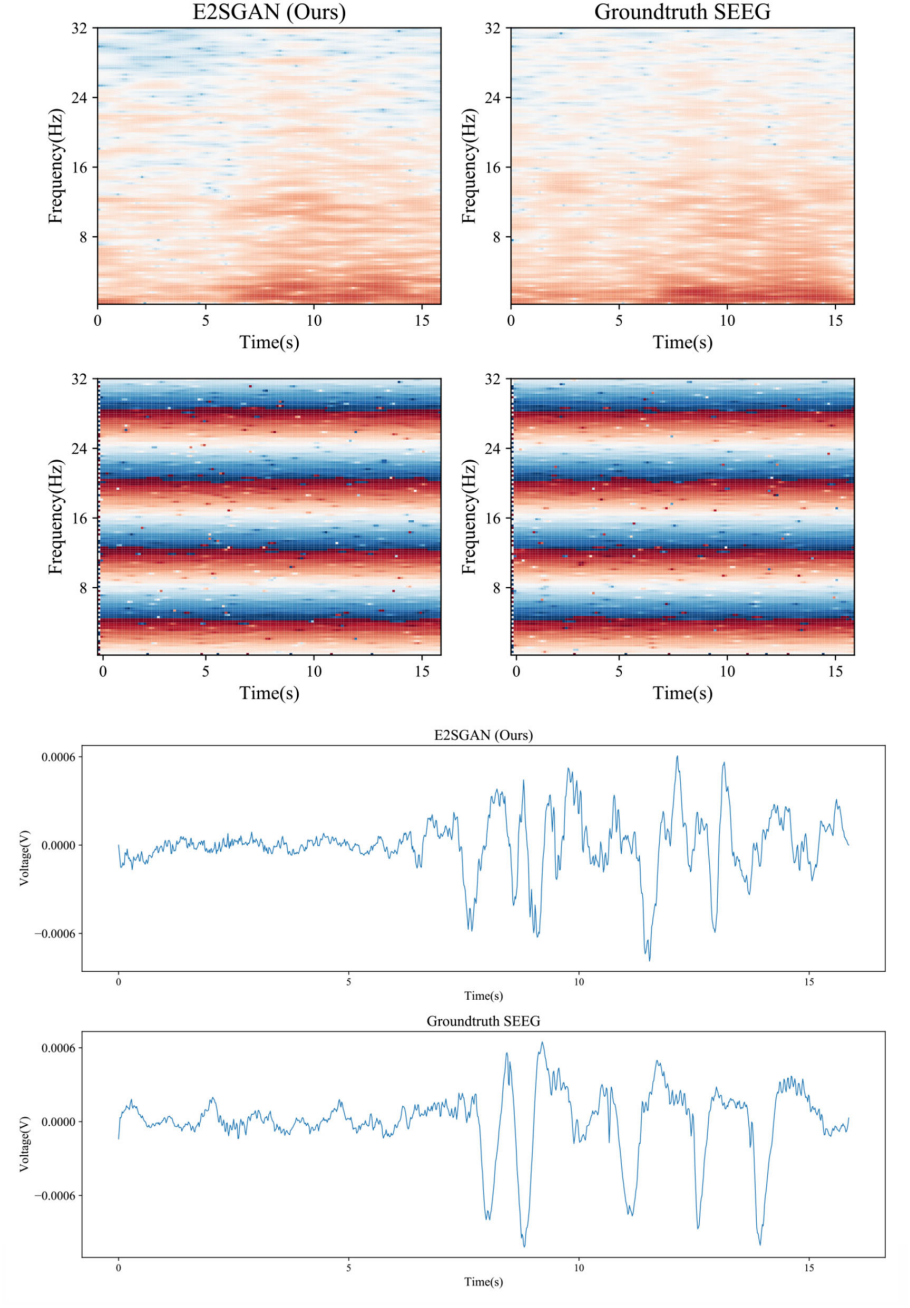
Case study of spectral results **(top)** and temporal results **(bottom)** synthesized by the proposed method compared to the groundtruth.

A visual case study is conducted by comparing the spectral and temporal results synthesized by the proposed method to the groundtruth. As is shown in the top rows of Figure 7, the proposed method produces clear results in both frequency and IF spectra, which indicates it has captured the details of how the power and phase are distributed along with time. From the bottom rows, it can be seen that the proposed method is able to produce the general morphology highly close to the groundtruth although the details of ripples are still far from satisfactory.

## 4. Discussion

In this paper, a GAN-based framework is introduced for the task of EEG-to-SEEG translation. First, a matching strategy is developed to select EEG-SEEG pairs. Second, E2SGAN is proposed to learn from the magnitude and IF spectra to synthesize the simultaneous SEEG segment given an input EEG segment. CSA and WPP technique are proposed to give a further boost to the discriminator. Extensive comparison experiments have demonstrated the capability of the proposed framework to transform EEG segments into SEEG segments. To find out whether the model has captured clinically significant features, a perturbation experiment is conducted. The final result shows that the synthesized SEEG signal retains the abnormal discharges before seizures.

### 4.1. Why not directly model the physical distance between EEG and SEEG?

Intuitively, it is more reasonable to generate the nearest EEG-SEEG pairs. We favor the impact of physical distance on SEEG generation, but it is not the only factor. In this work, we have tried to match EEG-SEEG pairs using a strategy based on the nearest physical distance. However, we found that EEG-SEEG pairs based solely on the physical distance were not necessarily strongly correlated (see Section 2.2 and Figure 3). This observation has also been confirmed in relevant clinical studies, where the geometry of the brain and its complex interconnections affect signal propagation (Frauscher et al., 2018). In the comparison experiment, we also experimented with the nearest physical distance matching strategy, but the results were not satisfactory. Therefore, we incorporated the HD distance into the matching strategy to limit the correlation between an EEG-SEEG pair. In this way, we avoid the influence of some unrelated pairs in the training process, even if they have a relatively close physical distance.

We agree that modeling the physical distance into the model is a worthwhile and practical consideration, and there have been many related works to spatially model the fixed position of EEG (Fang et al., 2020; Jia et al., 2021). However, for SEEG electrodes, this can be a huge challenge. SEEG positions are not fixed and cannot be uniformly modeled for all patients. Additionally, due to the very limited number of patients with simultaneous SEEG data we have access to, it is difficult to learn location representation with such large variance across different patients from a data-driven perspective. Another approach is to learn a fixed position representation and train a model per patient. Although this is feasible, the next problem we cannot avoid is that the position space of SEEG is continuous. For the generation task, we cannot achieve the goal of generating the accurate SEEG segment given an arbitrary position in the continuous space. To this end, the proposed matching strategy can essentially be viewed as discretizing the continuous position space (by dividing the physical distance into intervals) and selecting the “nearest” electrode pair that guarantees EEG-SEEG correlation. In other words, we incorporate physical distance as a prior knowledge into the data preprocessing process. In future work, we aim to collect a larger dataset and try to model the electrode position, distance, direction, and other information in a unified way through the deep-learning network to explore whether the existing technology can fully capture the rich clinical information.

### 4.2. Trade-off between extracted features and raw signal

When analyzing EEG signals, clinicians can judge whether there are signs of seizures through either the raw waveform and spectrum or the meaningful features computed by mathematical methods. Different input types affect the difficulty of generation tasks.

If the extracted features are used for the generation, the generated results can be directly applied to specific downstream tasks (Luo Y. et al., 2020). Traditional signal features such as Differential Entropy (DE) and PSD are highly informative and discriminative for specific tasks such as emotion recognition. They can be easily learned by neural networks and generate accurate results that are suitable for downstream tasks. However, the high compatibility with downstream tasks also limits the applicability of the generated results. For each feature or task, a neural network needs to be trained separately, which is not feasible in clinical use.

If the raw signal is directly used as the target, the generated results are applicable to a wider range of scenarios. Ideally, the generated results would approximate the real signal distribution, on which clinicians can perform any posterior analysis of interest. However, this type of generation is tricky because the informative signal components are often sparse and difficult for the network to fit.

There is a trade-off between using the extracted features or the raw signal as the generation target. Hence, large-scale simultaneously recorded EEG-SEEG datasets are necessary, which can improve the performance of end-to-end generation tasks to a certain extent. In addition, appending a downstream task to the generation stage that recognizes specific features can further improve the generation results, while ensuring good adaptability to specific tasks at the same time.

### 4.3. Challenges of different channel mapping assumptions

So far, only the one-to-one channel mapping has been considered in this work. Guided by this assumption, the proposed method can theoretically capture arbitrary EEG-SEEG correlations as long as the available dataset covers adequate EEG-SEEG combinations and achieve the brain-wide generative capability. In practice, this mapping assumption is not always plausible due to complex electrode arrangements. There are situations where it is impossible to find electrodes that fit the distance required by the proposed strategy, such as those positioned nearby wounds. Moreover, the scarcity of patients with synchronized EEG recordings is still a hindrance.

A more realistic assumption than one-to-one modeling is multiple-to-one/multiple-to-multiple mapping. EEG, as the collection of SEEG on the scalp, is actually the superposition of multiple intracranial discharge sources. However, multiple-to-one/multiple-to-multiple mapping is a more intractable problem that requires elaborate modeling techniques to extract the universal relations among multiple heterogeneous channels. For clinical research based on EEG signals, the study of multiple-to-multiple mapping is of great significance because the interpretive work based on it can reveal how intracranial signals propagate, thereby assisting clinicians to solve the problem of source localization. In the future, graph modeling using GCN may be more suitable for tasks based on this assumption, and graph-based interpretation algorithms will reveal richer principles of intracranial EEG signal propagation.

For future work, our proposed method can serve as a baseline for solutions developed on massive datasets or as a benchmark for formulating the multiple-to-one/multiple-to-multiple assumptions.

### 4.4. Unsupervised learning in SEEG generation

In this research, the adopted GANs framework is an unsupervised learning paradigm. Unsupervised learning eliminates the stage of labeling sample categories by experts and gets rid of the constraints of limited data sets. This property is particularly valuable because clinical data is often massive and unlabeled, and qualified clinicians rarely have the chance to withdraw from clinical work. With the advent of unsupervised learning, a new paradigm of pre-training has also emerged and has been applied in EEG research (Kostas et al., 2021; Yue et al., 2022; Zhang et al., 2022). This new paradigm inspired us to see if pre-trained models trained on large-scale EEG datasets can be used to generate SEEG signals after a fine-tuning stage. This is also one of the ideal solutions for the research topic in this paper.

## Data availability statement

The raw data supporting the conclusions of this article will be made available by the authors, without undue reservation.

## Ethics statement

The studies involving human participants were reviewed and approved by Institutional Review Board, Huashan Hospital, Fudan University. The patients/participants provided their written informed consent to participate in this study.

## Author contributions

MH, JC, WJ, and XW designed the proposed framework and drafted the manuscript. SJ, SM, and LC collected the data and analyzed the experiment results. All authors contributed critically to this work and approved of the upcoming edition.

## Funding

This work was supported by NSFC grants (No. 62136002 and 61972155) and the Science and Technology Commission of Shanghai Municipality (20DZ1100300).

## Conflict of interest

The authors declare that the research was conducted in the absence of any commercial or financial relationships that could be construed as a potential conflict of interest.

## Publisher's note

All claims expressed in this article are solely those of the authors and do not necessarily represent those of their affiliated organizations, or those of the publisher, the editors and the reviewers. Any product that may be evaluated in this article, or claim that may be made by its manufacturer, is not guaranteed or endorsed by the publisher.
