# Supplementary material for: E2SGAN: EEG-to-SEEG translation with generative adversarial networks
Source: Front Neurosci. 2022 Sep 1;16:971829. doi: 10.3389/fnins.2022.971829 (PMC9477431; doi:10.3389/fnins.2022.971829)
Supplement: Supplementary file 1 [file Presentation_1.PPTX]

## Slide 1
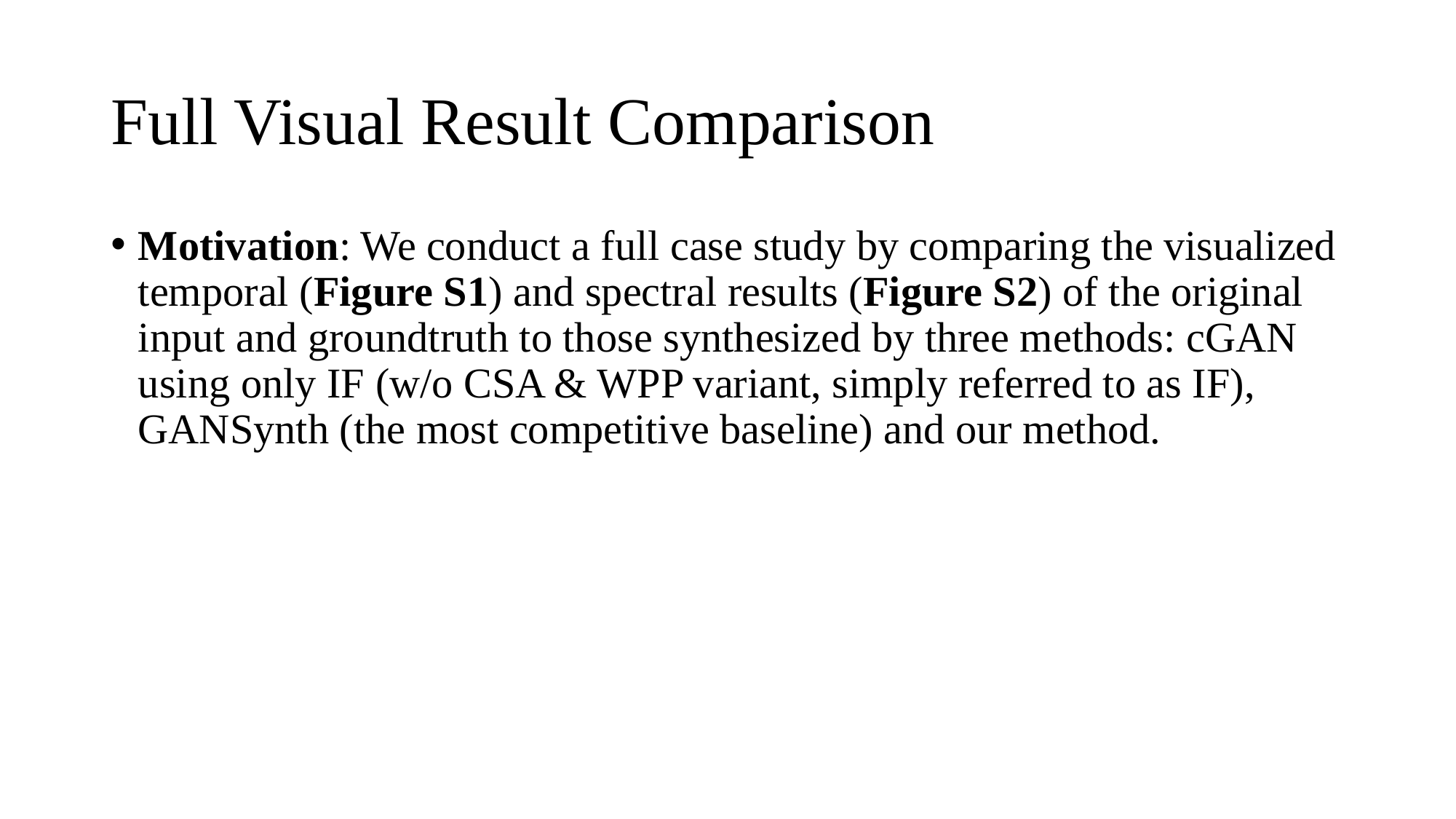

# Full Visual Result Comparison
Motivation: We conduct a full case study by comparing the visualized temporal (Figure S1) and spectral results (Figure S2) of the original input and groundtruth to those synthesized by three methods: cGAN using only IF (w/o CSA & WPP variant, simply referred to as IF), GANSynth (the most competitive baseline) and our method.

## Slide 2
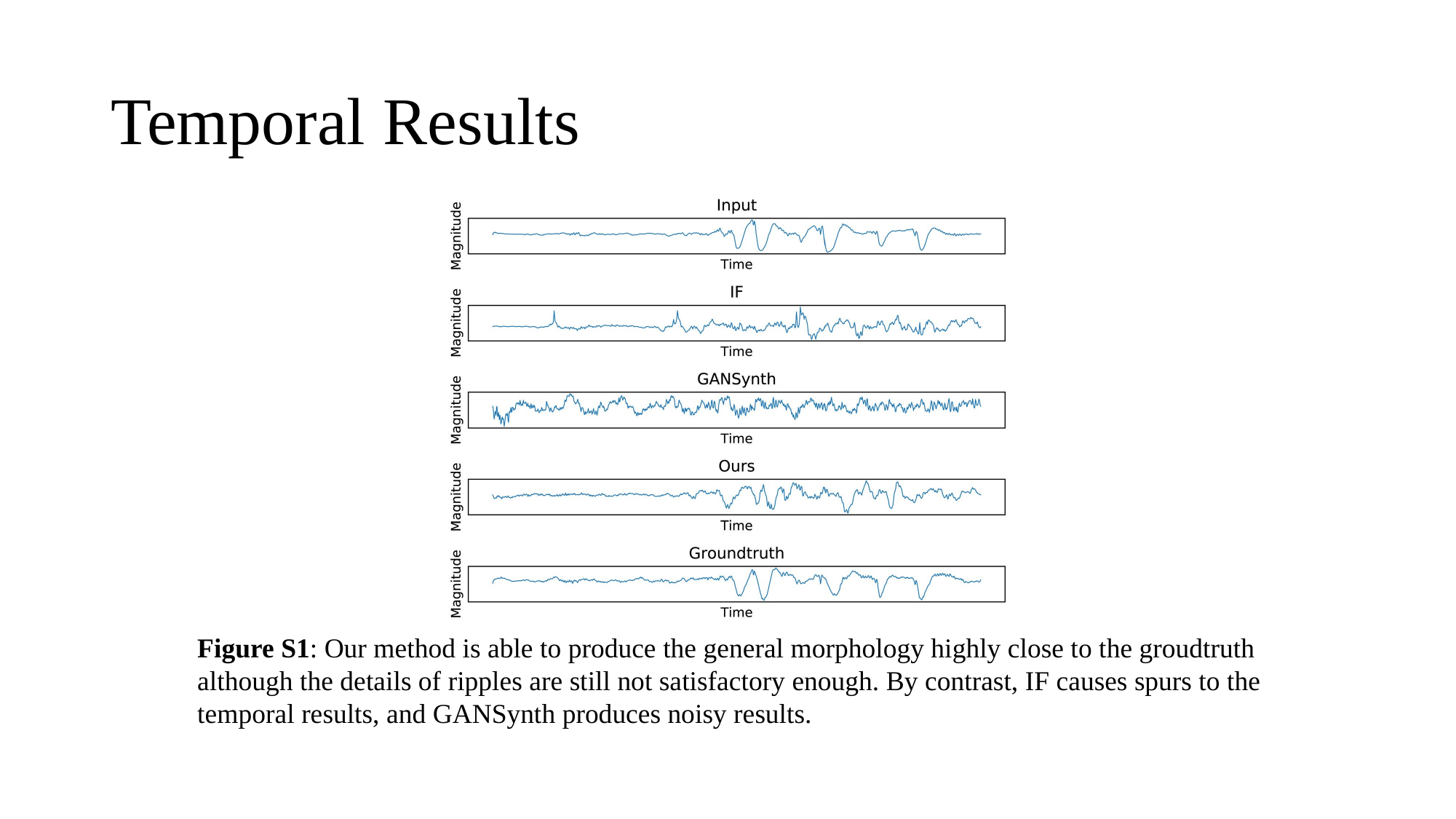

# Temporal Results
Figure S1: Our method is able to produce the general morphology highly close to the groudtruth although the details of ripples are still not satisfactory enough. By contrast, IF causes spurs to the temporal results, and GANSynth produces noisy results.

## Slide 3
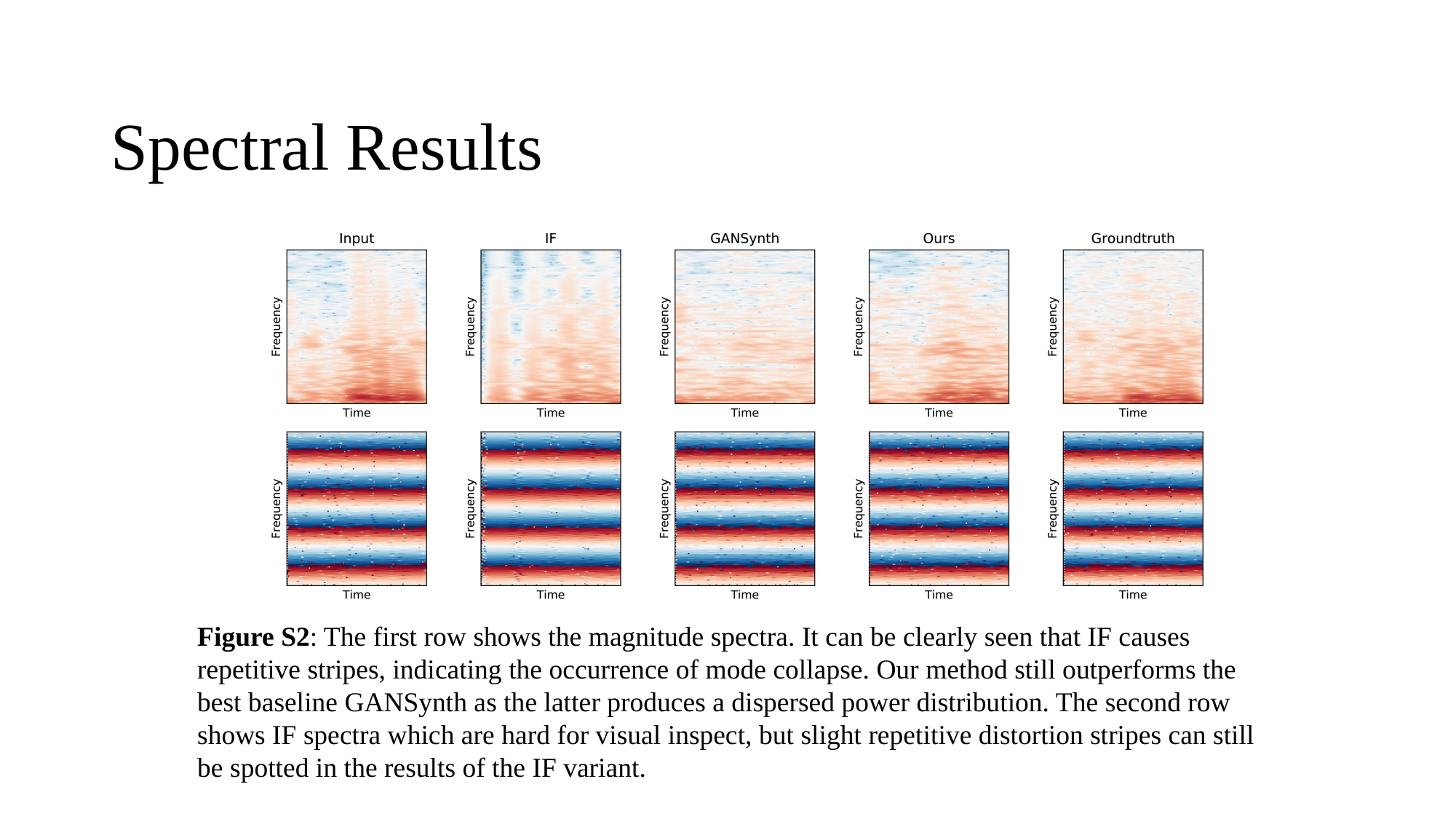

Spectral Results
Figure S2: The first row shows the magnitude spectra. It can be clearly seen that IF causes repetitive stripes, indicating the occurrence of mode collapse. Our method still outperforms the best baseline GANSynth as the latter produces a dispersed power distribution. The second row shows IF spectra which are hard for visual inspect, but slight repetitive distortion stripes can still be spotted in the results of the IF variant.

## Slide 4
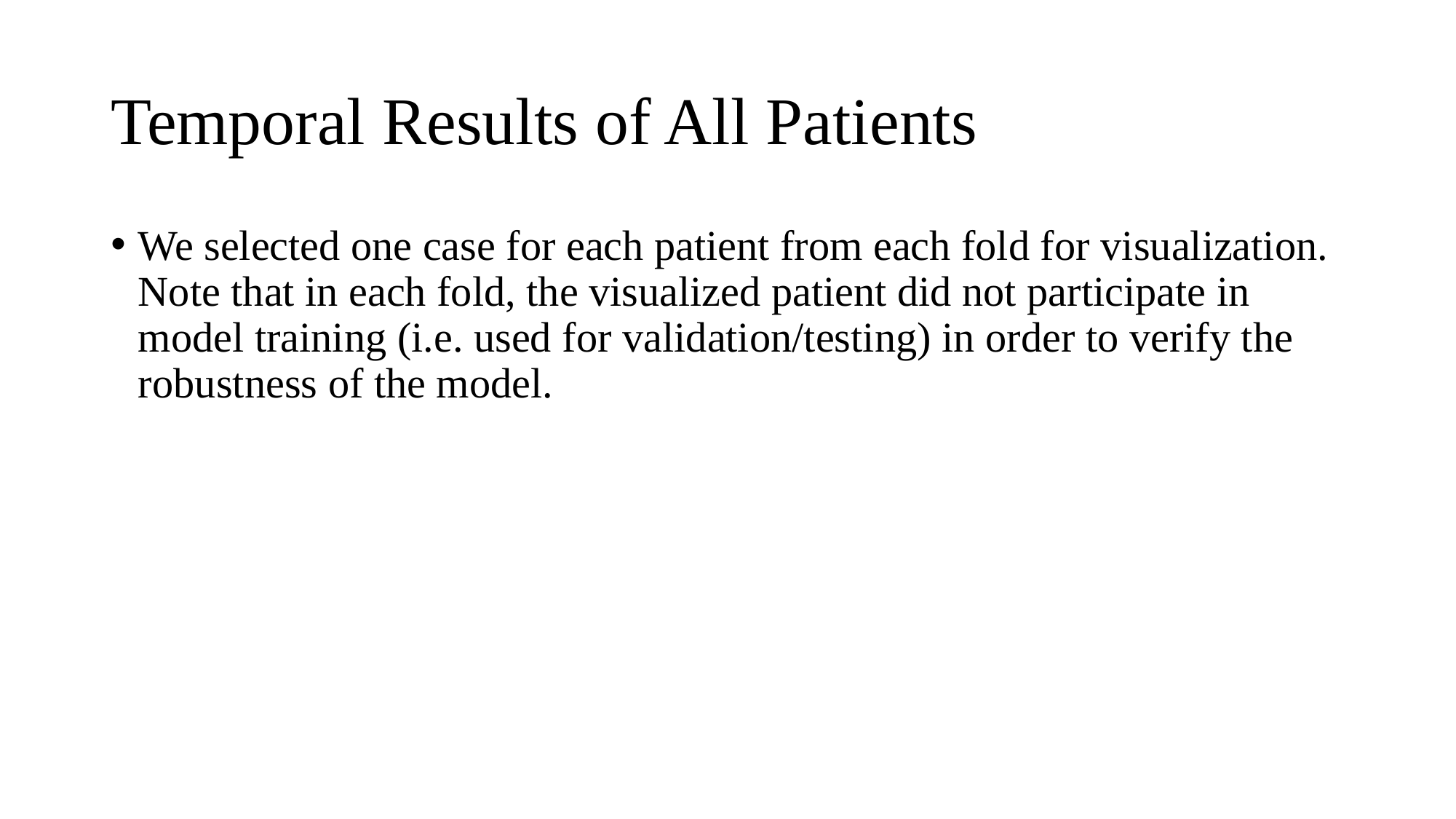

# Temporal Results of All Patients
We selected one case for each patient from each fold for visualization. Note that in each fold, the visualized patient did not participate in model training (i.e. used for validation/testing) in order to verify the robustness of the model.

## Slide 5
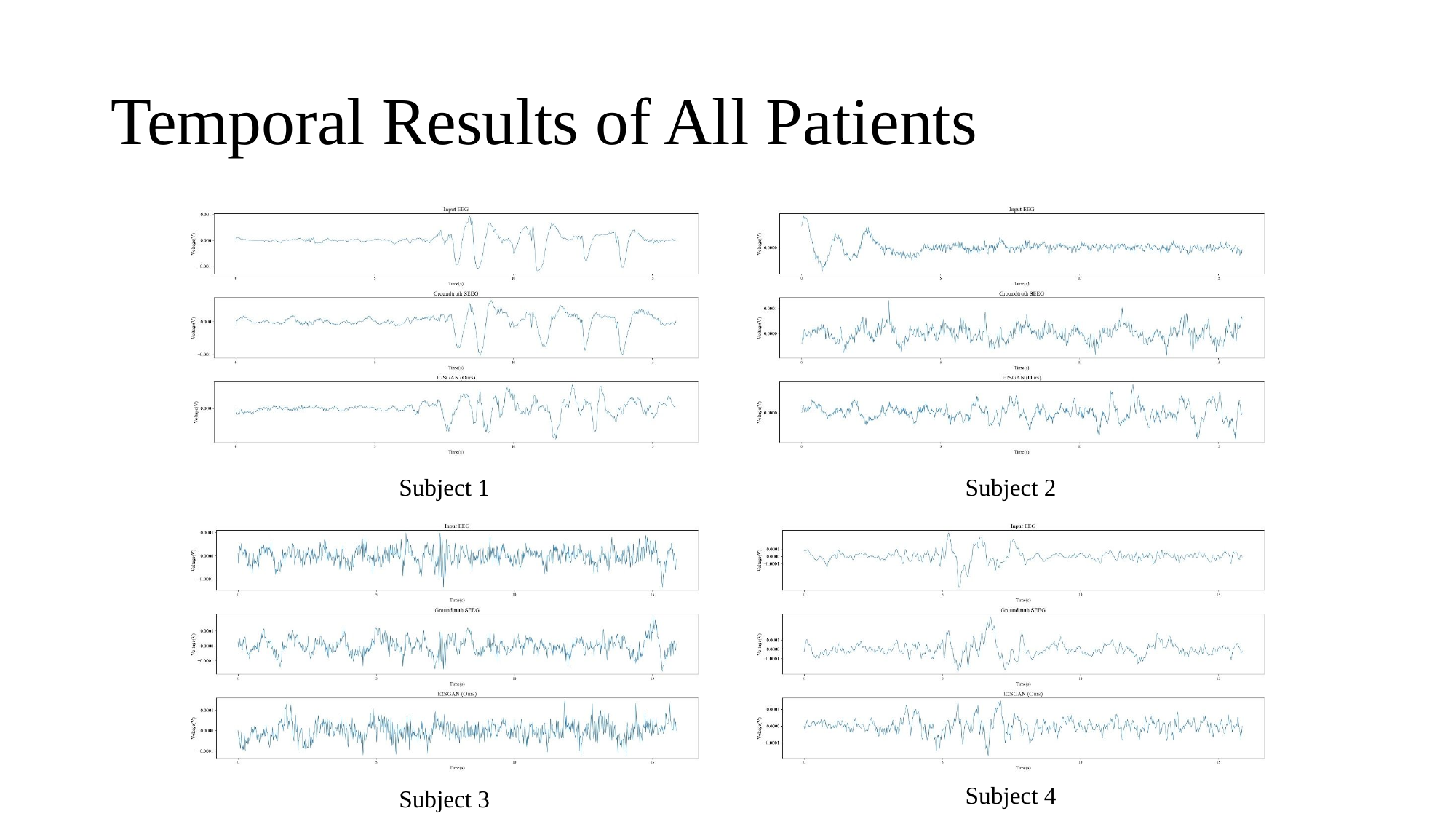

# Temporal Results of All Patients
Subject 1
Subject 2
Subject 4
Subject 3

## Slide 6
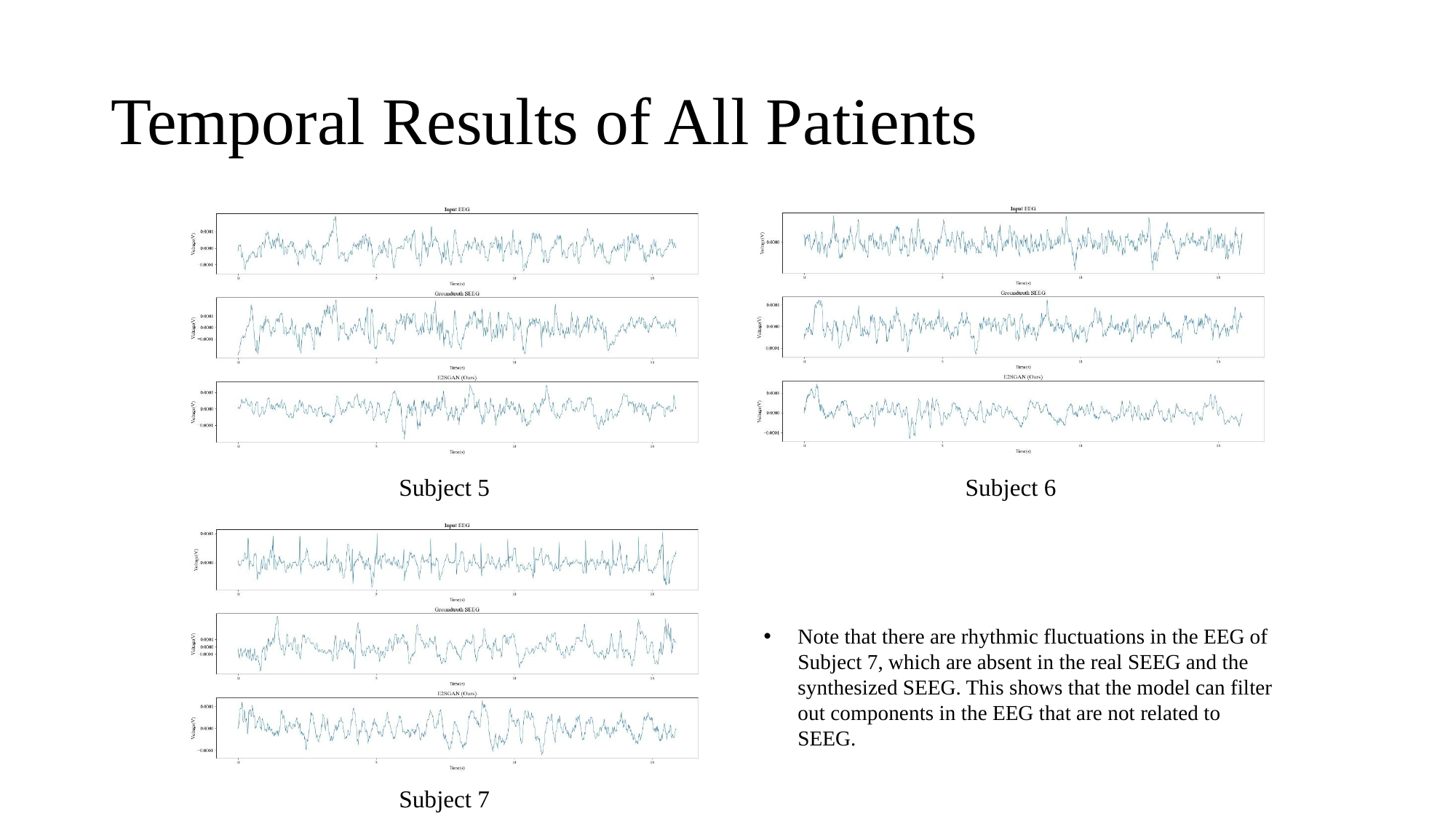

# Temporal Results of All Patients
Subject 5
Subject 6
Note that there are rhythmic fluctuations in the EEG of Subject 7, which are absent in the real SEEG and the synthesized SEEG. This shows that the model can filter out components in the EEG that are not related to SEEG.
Subject 7

## Slide 7
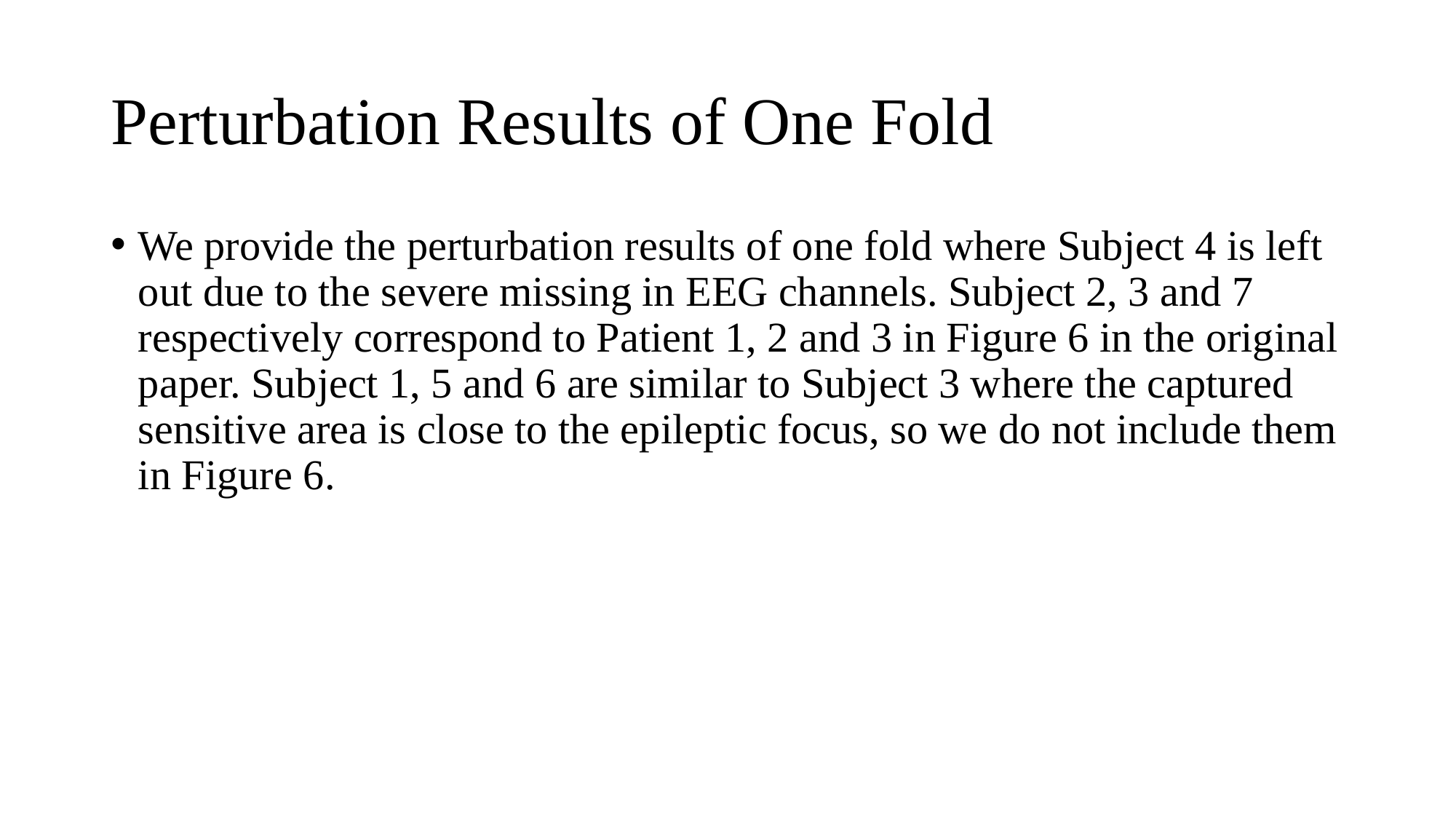

# Perturbation Results of One Fold
We provide the perturbation results of one fold where Subject 4 is left out due to the severe missing in EEG channels. Subject 2, 3 and 7 respectively correspond to Patient 1, 2 and 3 in Figure 6 in the original paper. Subject 1, 5 and 6 are similar to Subject 3 where the captured sensitive area is close to the epileptic focus, so we do not include them in Figure 6.

## Slide 8
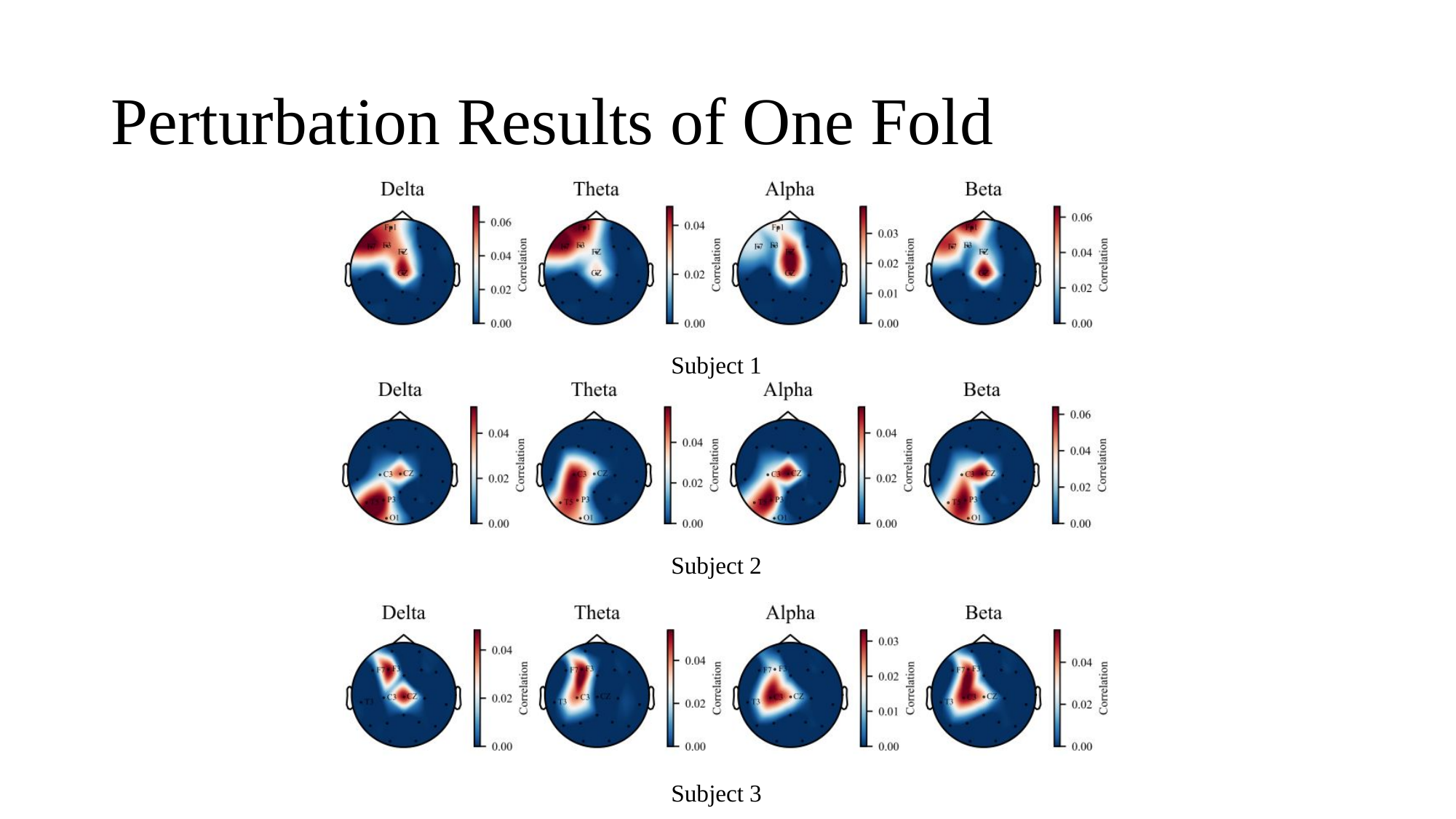

# Perturbation Results of One Fold
Subject 1
Subject 2
Subject 3

## Slide 9
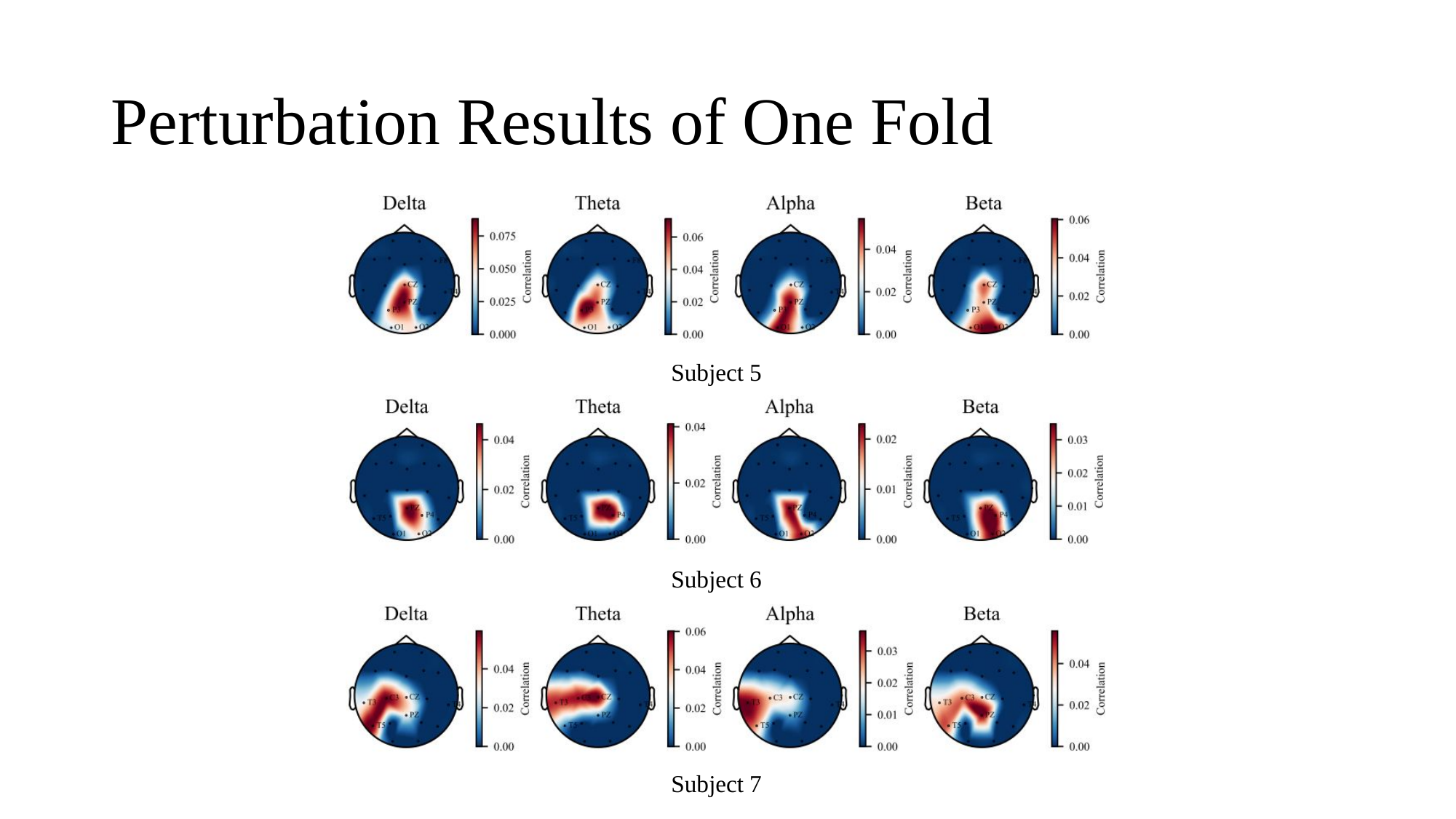

# Perturbation Results of One Fold
Subject 5
Subject 6
Subject 7

## Slide 10
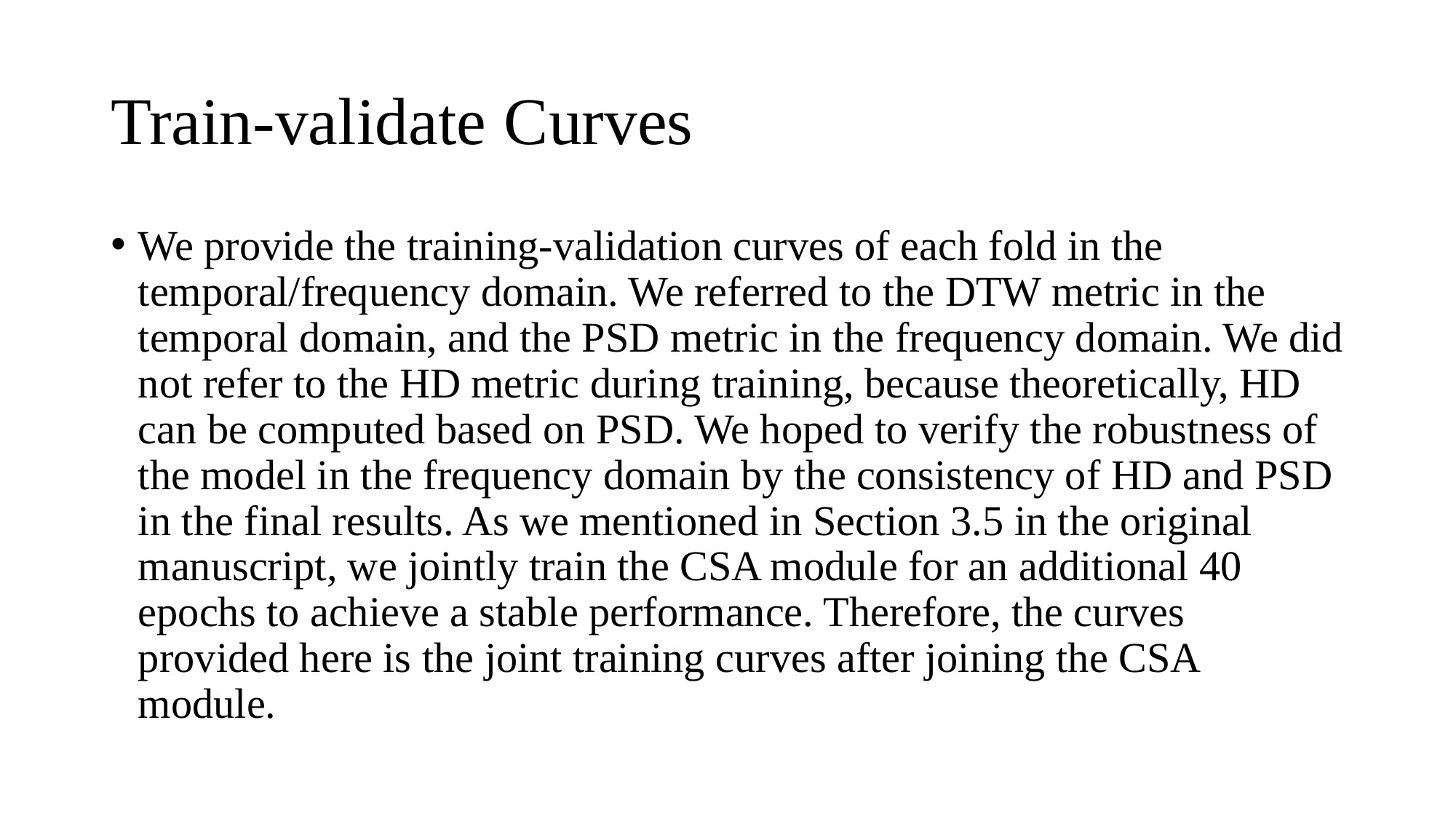

# Train-validate Curves
We provide the training-validation curves of each fold in the temporal/frequency domain. We referred to the DTW metric in the temporal domain, and the PSD metric in the frequency domain. We did not refer to the HD metric during training, because theoretically, HD can be computed based on PSD. We hoped to verify the robustness of the model in the frequency domain by the consistency of HD and PSD in the final results. As we mentioned in Section 3.5 in the original manuscript, we jointly train the CSA module for an additional 40 epochs to achieve a stable performance. Therefore, the curves provided here is the joint training curves after joining the CSA module.

## Slide 11
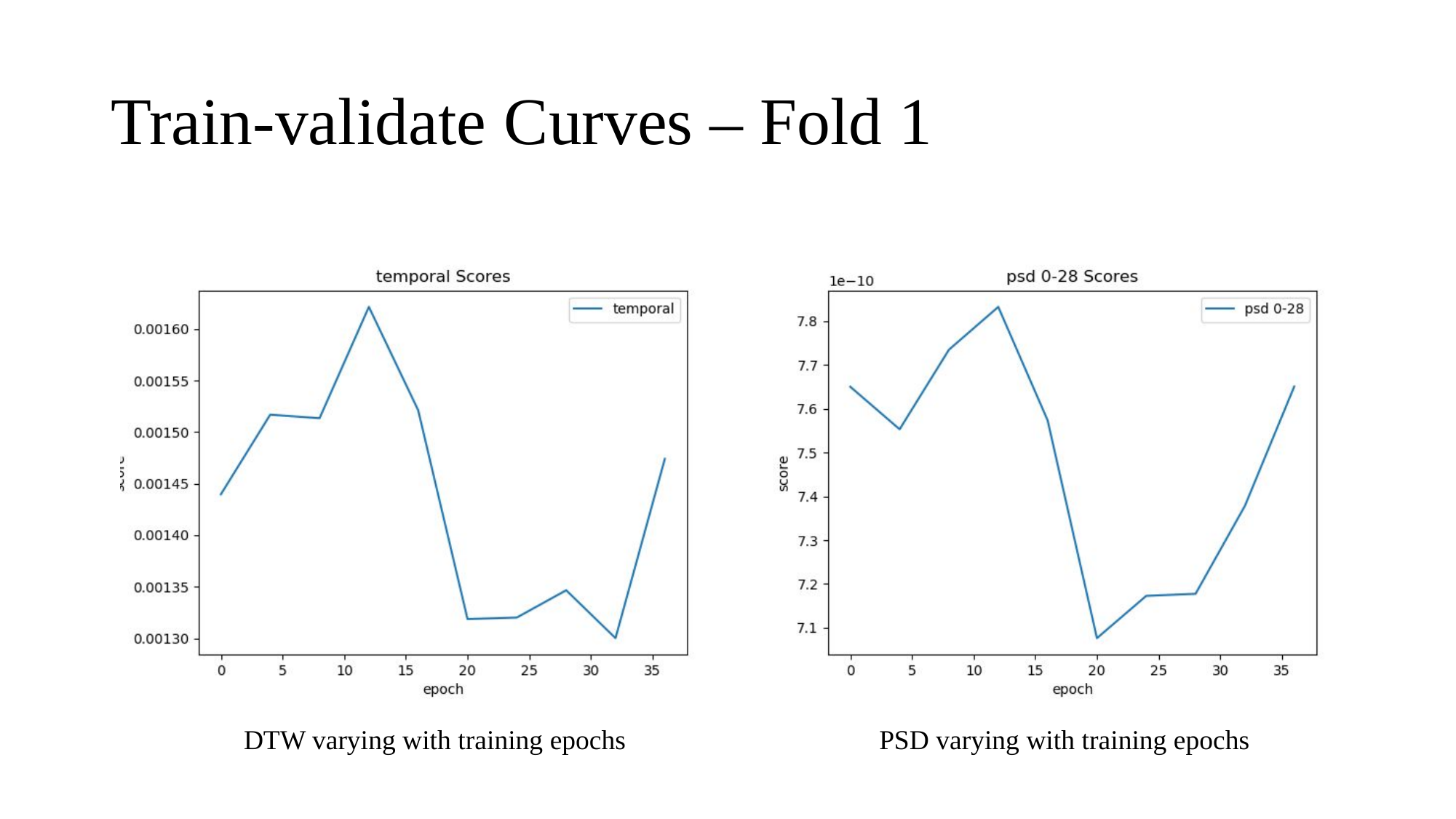

# Train-validate Curves – Fold 1
DTW varying with training epochs
PSD varying with training epochs

## Slide 12
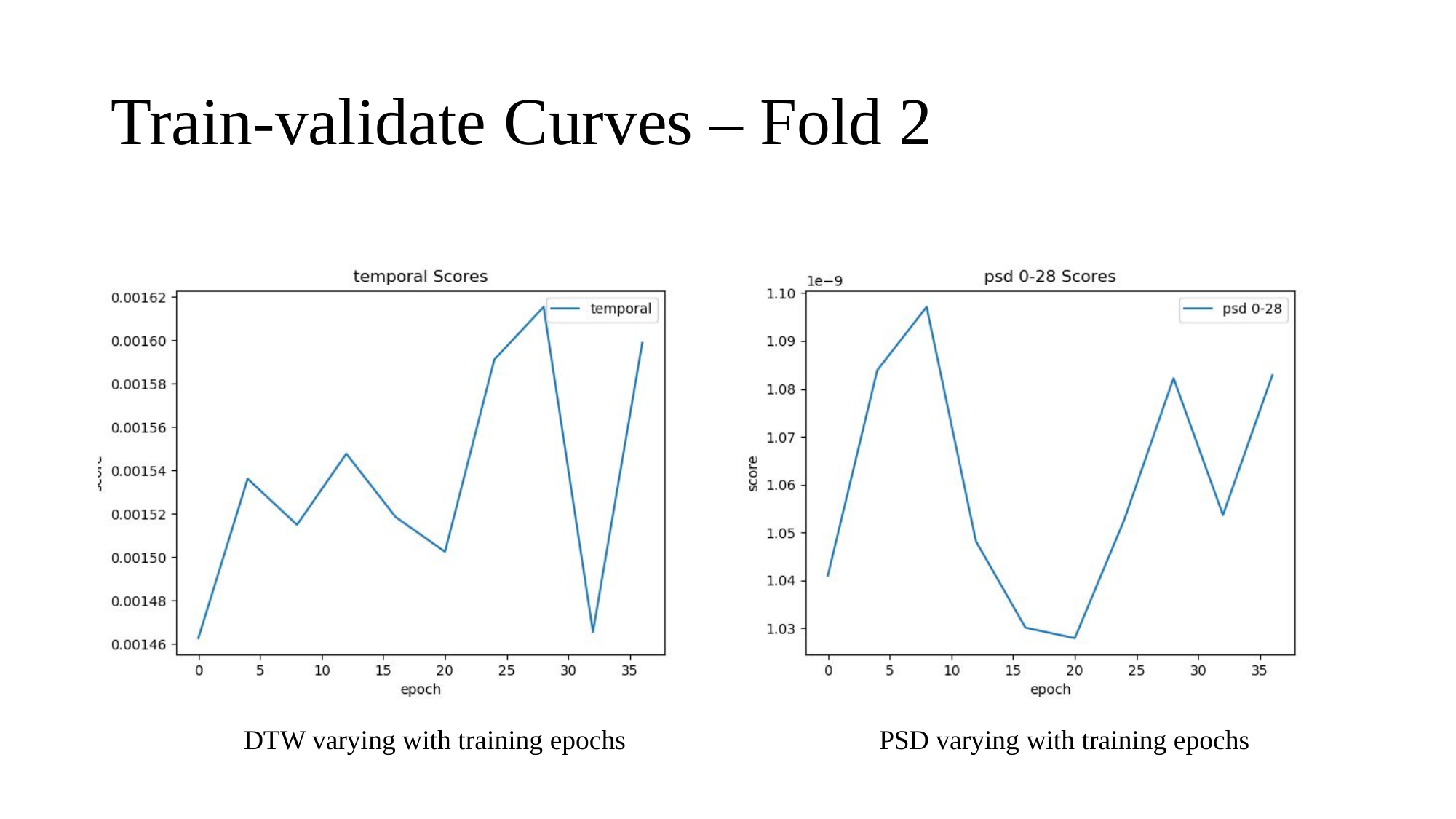

# Train-validate Curves – Fold 2
DTW varying with training epochs
PSD varying with training epochs

## Slide 13
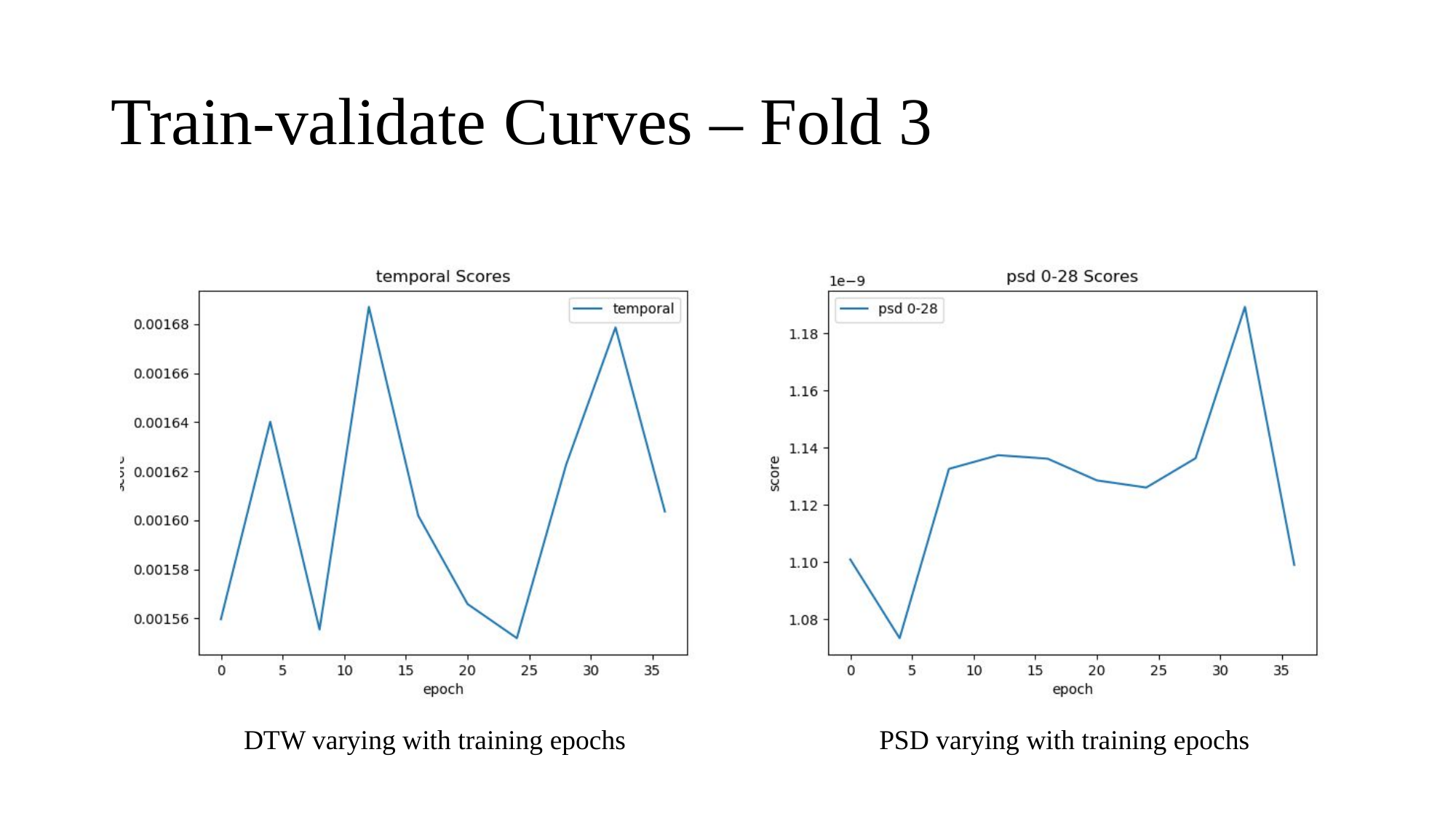

# Train-validate Curves – Fold 3
DTW varying with training epochs
PSD varying with training epochs

## Slide 14
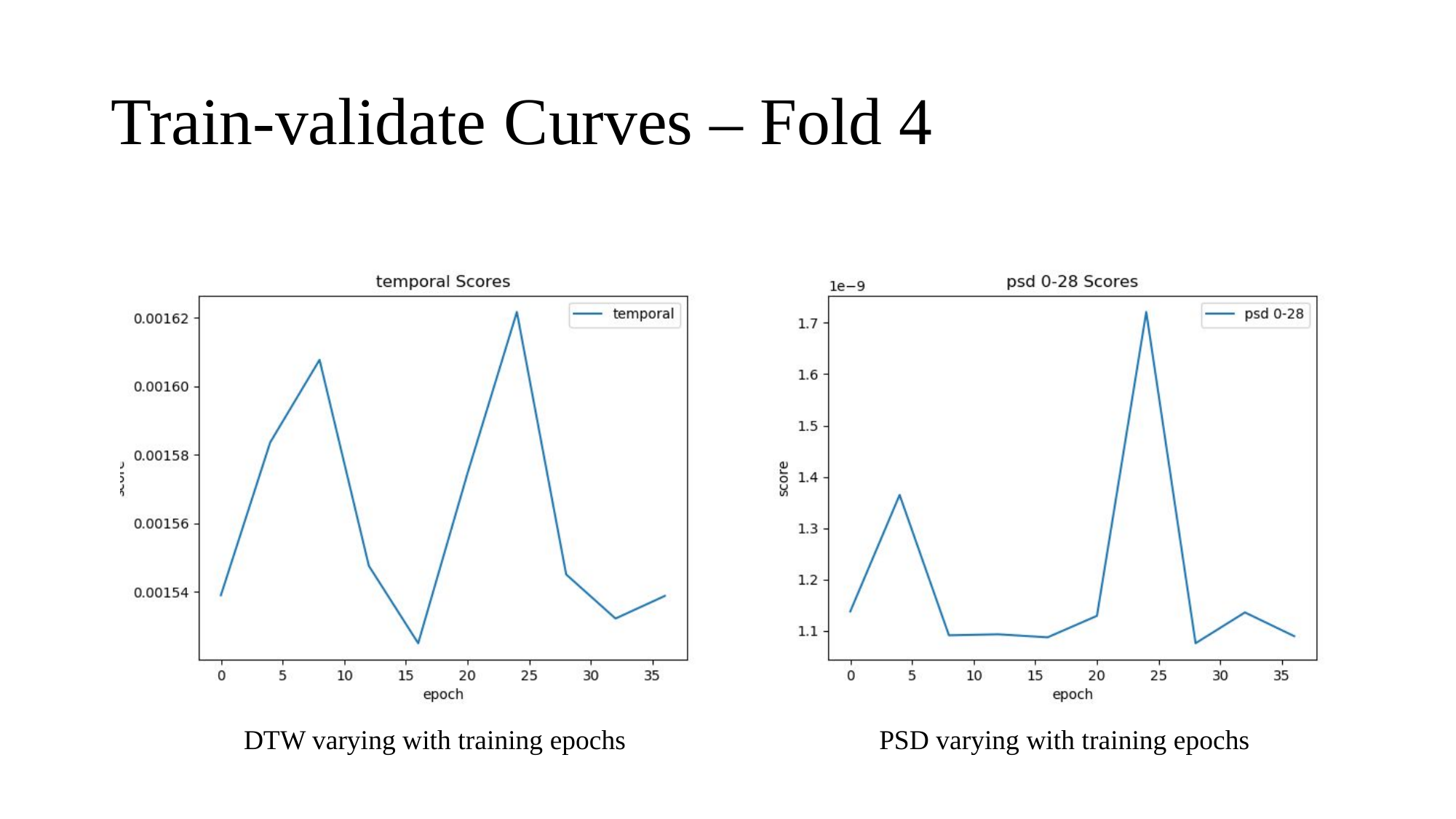

# Train-validate Curves – Fold 4
DTW varying with training epochs
PSD varying with training epochs

## Slide 15
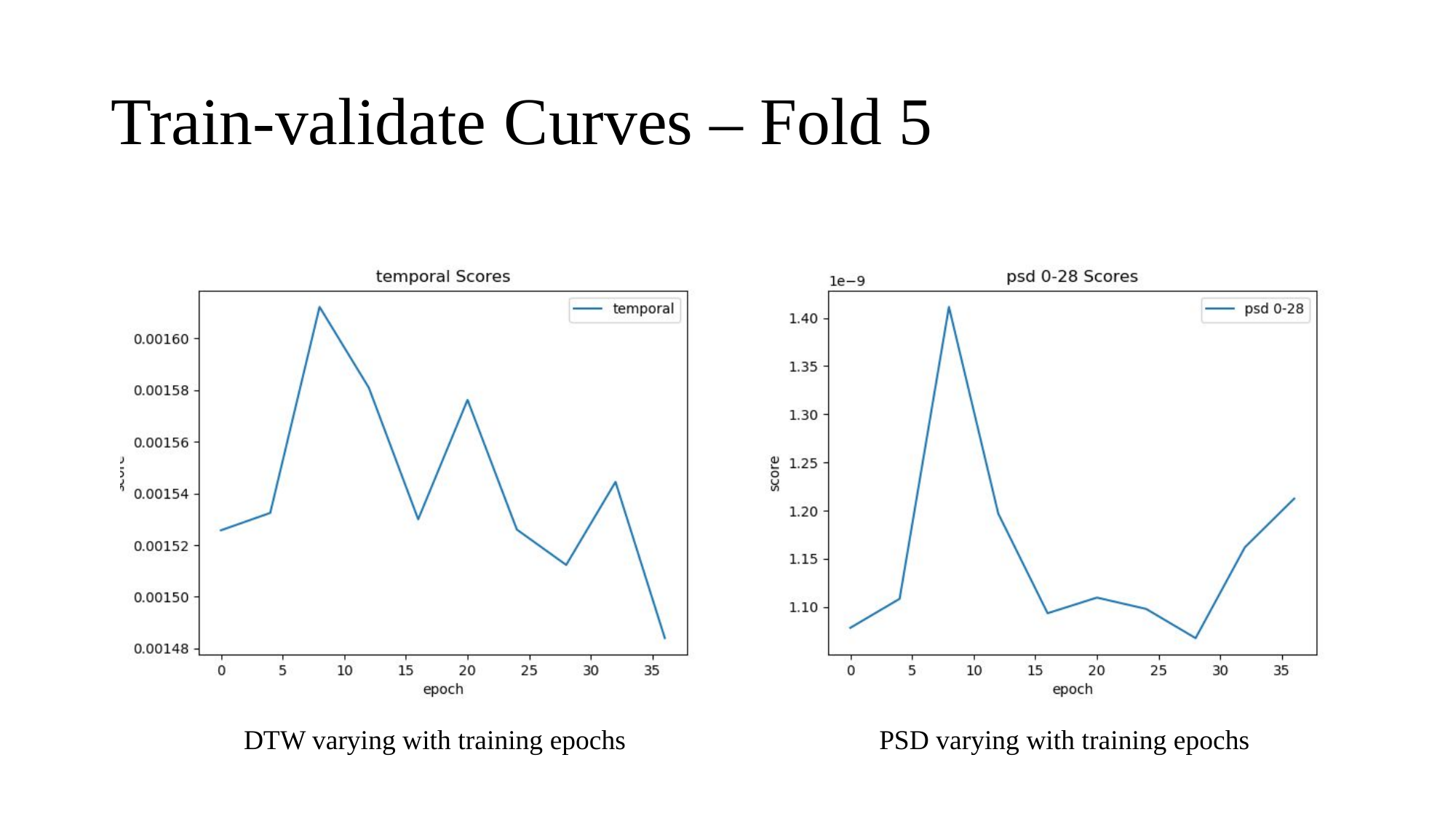

# Train-validate Curves – Fold 5
DTW varying with training epochs
PSD varying with training epochs

## Slide 16
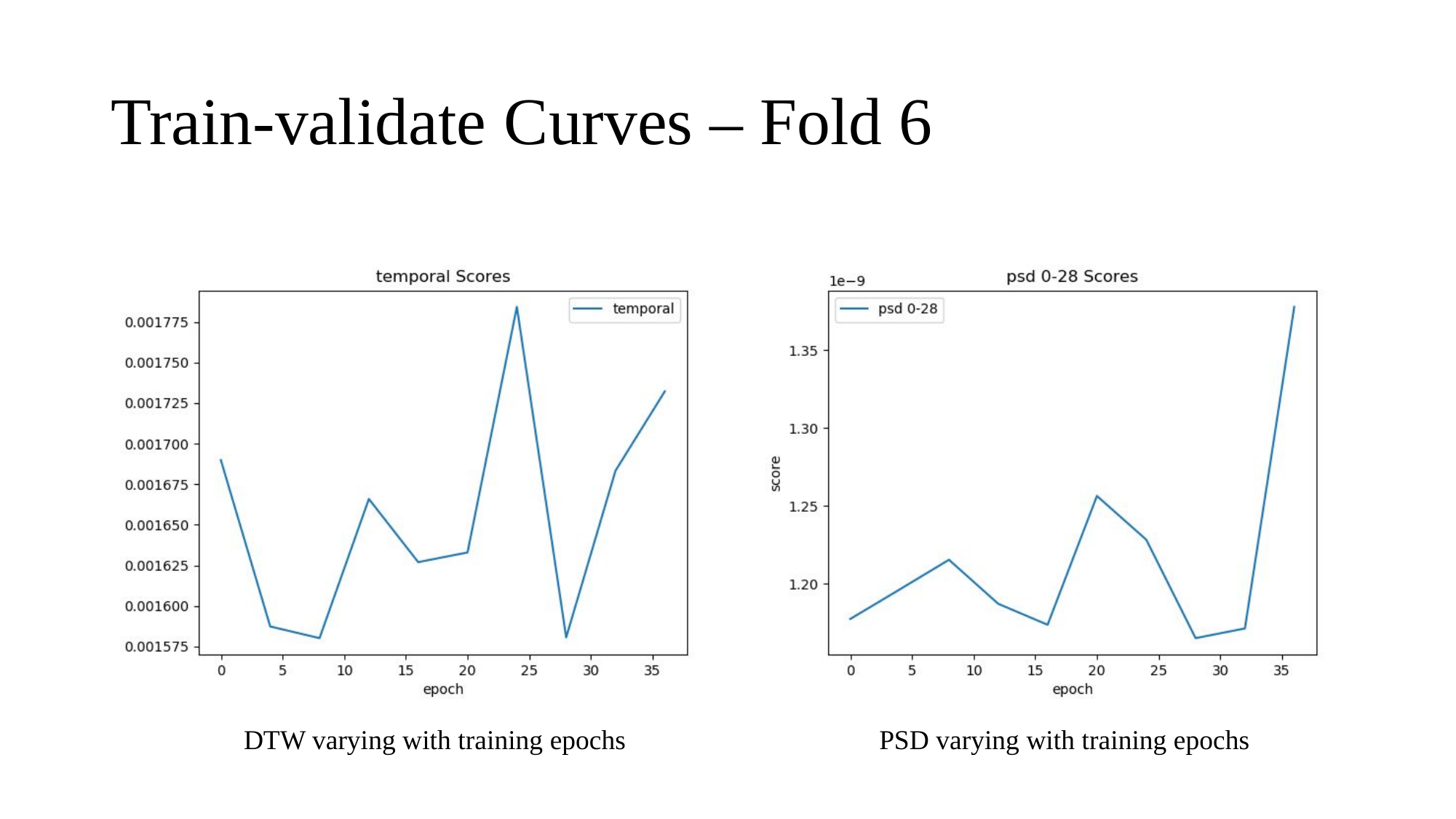

# Train-validate Curves – Fold 6
DTW varying with training epochs
PSD varying with training epochs

## Slide 17
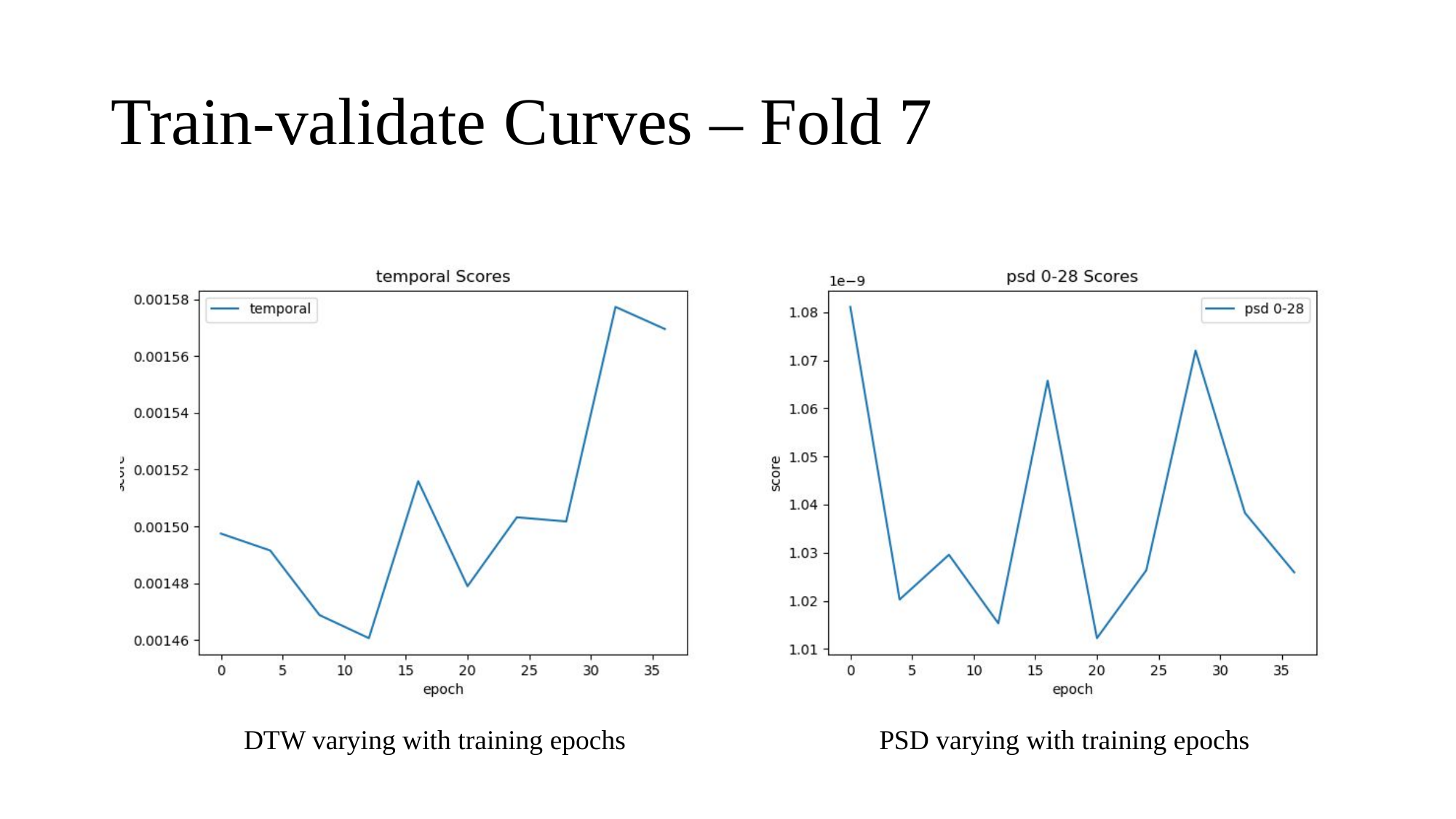

# Train-validate Curves – Fold 7
DTW varying with training epochs
PSD varying with training epochs

## Slide 18
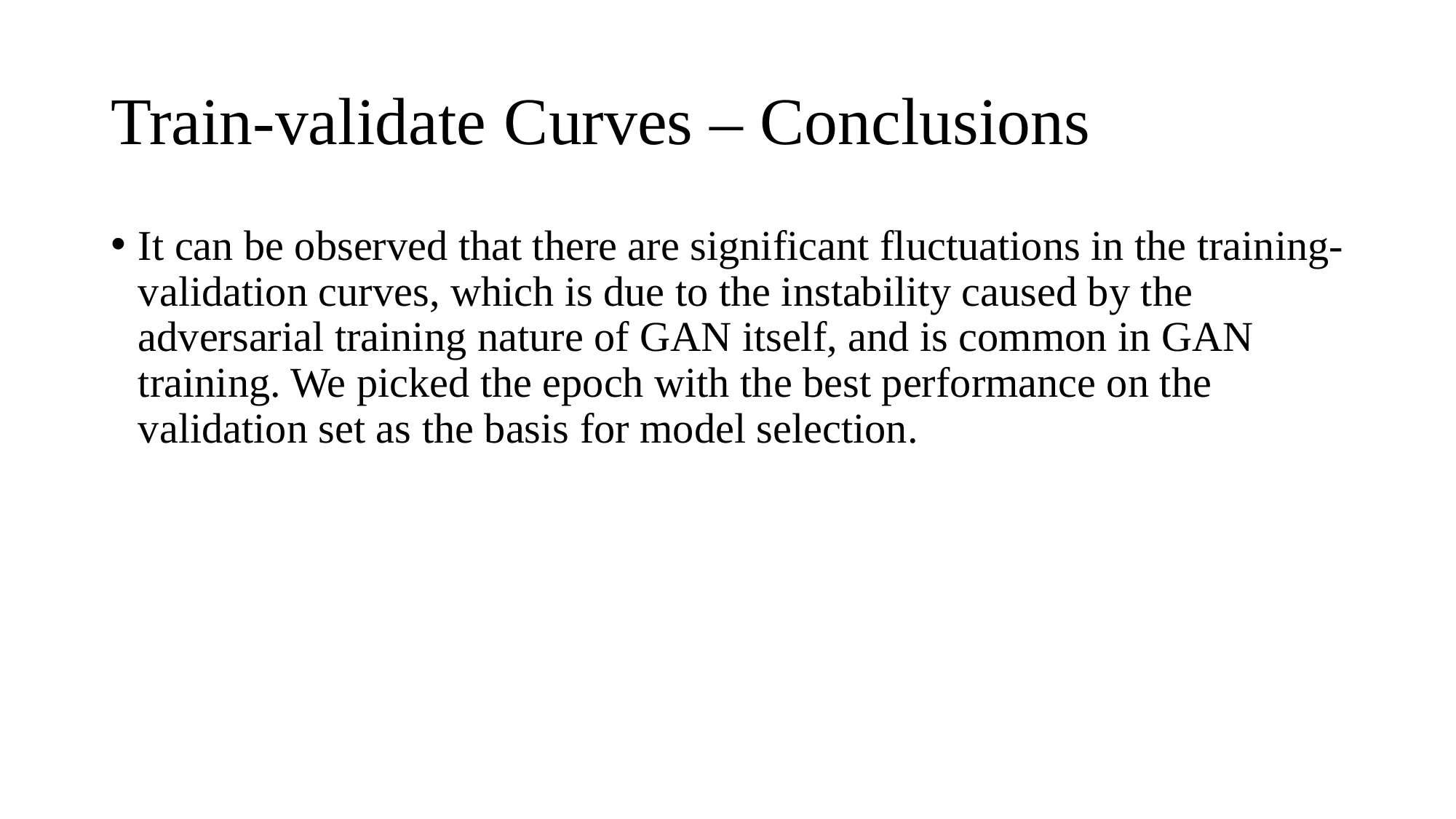

# Train-validate Curves – Conclusions
It can be observed that there are significant fluctuations in the training-validation curves, which is due to the instability caused by the adversarial training nature of GAN itself, and is common in GAN training. We picked the epoch with the best performance on the validation set as the basis for model selection.
